# Vertebrate Cryptochromes are Vestigial Flavoproteins

**DOI:** 10.1038/srep44906

**Published:** 2017-03-20

**Authors:** Roger J. Kutta, Nataliya Archipowa, Linus O. Johannissen, Alex R. Jones, Nigel S. Scrutton

**Affiliations:** 1 Manchester Institute of Biotechnology (MIB) and School of Chemistry, The University of Manchester, 131 Princess Street, Manchester, M1 7DN, UK; 2 Photon Science Institute and School of Chemistry, The University of Manchester, Alan Turing Building, Oxford Road, Manchester, M13 9PL, UK

## Abstract

All cryptochromes are currently classified as flavoproteins. In animals their best-described role is as components of the circadian clock. This circadian function is variable, and can be either light-dependent or -independent; the molecular origin of this difference is unknown. Type I animal cryptochromes are photoreceptors that entrain an organism’s clock to its environment, whereas Type II (including mammals) regulate circadian timing in a light-independent manner. Here, we reveal that, in contrast to Type I, Type II animal cryptochromes lack the structural features to securely bind the photoactive flavin cofactor. We provide a molecular basis for the distinct circadian roles of different animal cryptochromes, which also has significant implications for the putative role of Type II cryptochromes in animal photomagnetoreception.

Circadian rhythms manifest in almost all organisms and control a wide range of physiological processes. The endogenous clock achieves a roughly 24 h period through a network of transcriptional/translational feedback loops, and is entrained to the ambient conditions by cues such as light^1^. Disruption of normal circadian timing is associated with a variety of disease outcomes including cancer and diabetes^2^ and recruitment of immune cells to tissues is under circadian control^3^. Cryptochromes (CRY) are clock proteins that act as circadian regulators throughout nature^4^, and which have a putative role in animal magnetoreception^5^,^6^,^7^,^8^. In a circadian context, there are two types of animal CRY. Type I animal CRY, found in invertebrates such as *Drosophila melanogaster*, are primary blue-light photoreceptors that entrain the organism’s circadian clock to the light/dark cycle of their environment^9^,^10^,^11^. By contrast, Type II CRY found in vertebrates as well as in invertebrates^12^,^13^ are transcriptional repressors in the negative feedback loops of the central and peripheral clocks^14^,^15^. Mammalian Type II CRY are known to couple the circadian clock to metabolism through interaction with glucocorticoid receptors^16^. The circadian functions of Type II CRY appear to occur independently of light^17^.

The molecular basis of the difference in biological function between Type I and II animal CRY is a major unanswered question in circadian biology. Type I, photoreceptor CRY from *Drosophila melanogaster* (DmCRY) binds the blue-light chromophore and redox active cofactor, flavin adenine dinucleotide (FAD), in its fully oxidized form^18^,^19^,^20^,^21^. Much like the closely related photolyases^22^, FAD is bound to DmCRY in an unusual U-shape, with the adenine in close proximity to the isoalloxazine. Type II CRY, although assumed to be flavoproteins, have been notoriously difficult to express and isolate with bound FAD^19^,^23^,^24^,^25^,^26^,^27^,^28^. We therefore hypothesize that a major contributing factor to the contrasting roles of Type I and Type II animal CRY is the differential binding of FAD. Such differential binding is likely to act in concert with, or even directly influence, other important factors such as the different interaction partners and genetic networks that exist within the clock of invertebrates and vertebrates. To test this hypothesis, we conducted a combined experimental/computational study of FAD-binding to typical Type I and Type II CRY.

## Results

### Type II cryptochromes are expressed without bound FAD

**Figure 1:**
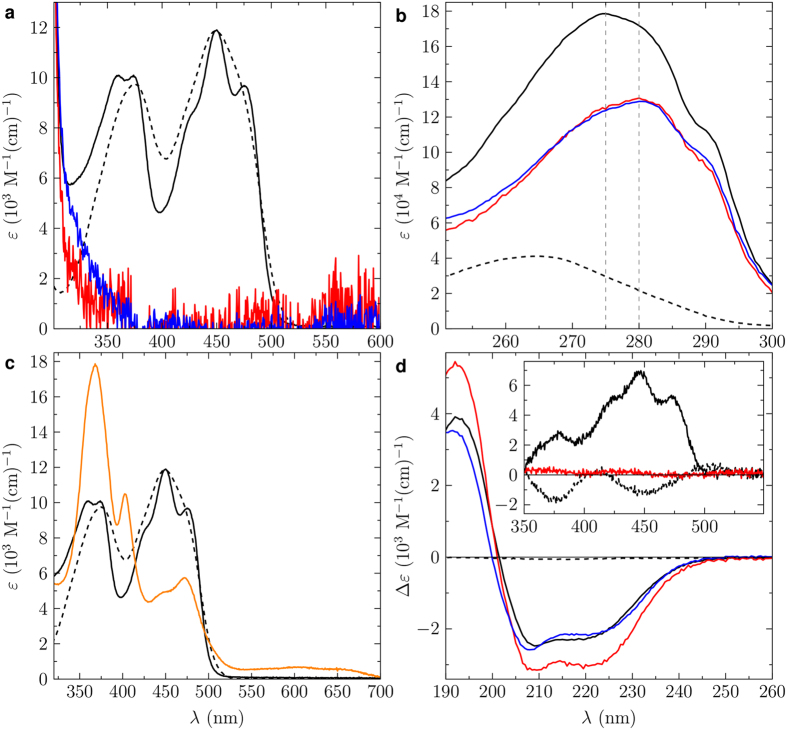
Absorption and circular dichroism spectra of typical Type I and Type II CRY. (**a**) UV-visible absorption spectra of free FAD (black dashed), DmCRY (black), HsCRY1 (red), and HsCRY2 (blue). (**b**) Magnification of the absorption band at 280 nm from (**a**). (**c**) UV-visible absorption spectra of free FAD, DmCRY ‘dark’ (black) and ‘light’ (orange) state (see also Fig. S2). (**d**) CD spectra in the UV range. Inset: CD spectra in the visible range (colour coding as in (**a**)).

We expressed and purified recombinant DmCRY (Type I) and *Homo sapiens* HsCRY1/2 (Type II) from *Escherichia coli* (Fig. S1). The UV-visible absorption spectrum of DmCRY shows near-stoichiometric binding of oxidized FAD (Fig. 1a), with characteristic vibrational fine structure on the absorption bands around 350 and 450 nm^18^ (not present in the spectrum of unbound FAD), and a 5 nm blue-shift of the 280 nm absorption band (Fig. 1b). DmCRY also forms a stable FAD semiquinone radical after blue light illumination attributed to electron transfer from aromatic residues in the protein to the FAD isoalloxazine (Figs 1c and S2). By contrast, no FAD is evident in the UV-visible absorption spectra of HsCRY1 and 2 (Fig. 1a,b). UV circular dichroism (CD) spectra of DmCRY and HsCRY1/2 show that all three proteins appear to be well-folded (Fig. 1d). Furthermore, the CD spectrum of DmCRY in the visible region shows a strong FAD signal, which is diagnostic of the flavin being bound in the chiral environment of the protein (Fig. 1d, inset). We confirmed the lack of flavin binding to HsCRY1/2 by incubation of each protein in excess FAD and removal of free FAD afterwards by spin filtration; no equivalent CD signal was observed. Furthermore, we quantified the FAD binding affinity *via* titration of each apo-protein to a constant concentration of free FAD and measuring the change in the UV-visible absorption spectrum due to FAD binding. We determined dissociation constants (*K*_D_) of 16 μM and 68 μM for HsCRY1 and 2, respectively (see Figs S3 and S4). This low FAD binding affinity explains why HsCRY1 and 2 are purified without FAD, and why the FAD could be washed off from the samples that were overloaded with FAD. In the case where FAD is bound to either HsCRY1 or 2, photo-induced reactivity between excited FAD isoalloxazine and aromatic amino acids of the protein backbone can be observed resulting in the formation of the neutral FAD semiquinone radical and the counter neutral tryptophan radical (see Figs S5 and S6). Interestingly, we were also able to observe its formation due to protonation of the initially formed anionic FAD semiquinone radical occurring with a lifetime of either 219 ms or 510 ms (see Figs S7a,b and S8a,b) prior to the decay into the fully oxidised FAD isoalloxazine within *ca*. 200s or *ca*. 50s (see Figs S7c,d and S8c,d) for HsCRY1 and 2, respectively. These results contrast with our previous report of HsCRY1 isolated after heterologous expression in *Pichia pastoris*, where FAD was ostensibly bound as a stable anionic semiquinone^27^. We have since discovered by mass spectrometry that HsCRY1 co-migrates during purification from *Pichia pastoris* with alcohol oxidase. This octameric enzyme native to yeast binds FAD in mixed oxidation states, but predominantly as the stable semiquinone, and the monomer is of a very similar mass (~70 kDa) to HsCRY1^29^.

### Computer simulations reveal low FAD binding affinity in Type II cryptochromes

**Figure 2:**
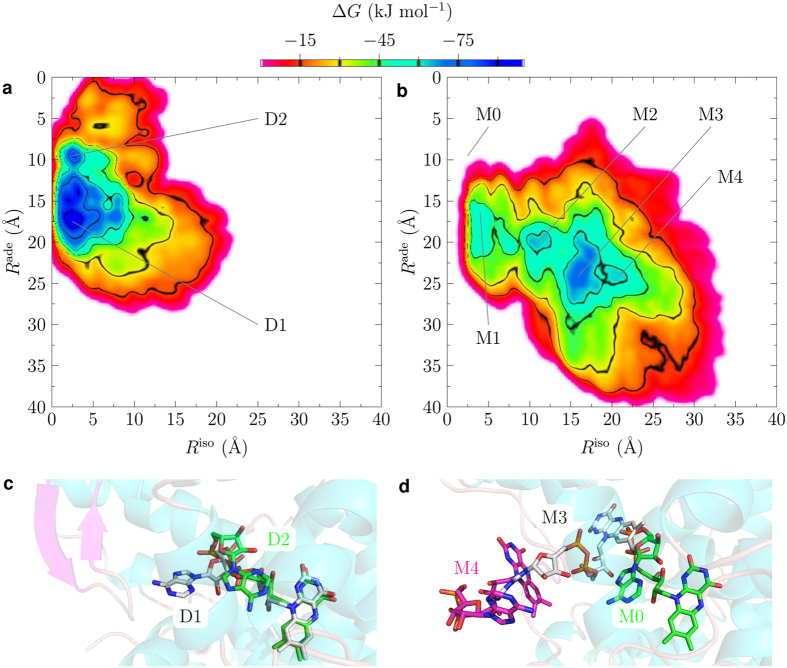
Metadynamics simulations of typical Type I and Type II CRY for FAD affinity. 2-dimensional free energy (Δ*G*) plots for FAD (un)binding from MTD simulations for DmCRY (**a**) and MmCRY2 (**b**) based on the average of five individual runs each. *R*^ade^ is the distance between the centre-of-masses of the adenine and the binding pocket residues, and *R*^iso^ the distance between the centre-of-masses of the isoalloxazine and the binding pocket residues. For exact definition of distances see Supplementary Materials. (**c**,**d**) Example structures from the indicated minima.

To further investigate the affinity for FAD of animal CRY we performed molecular dynamic (MD) and metadynamics (MTD) simulations^30^ using published CRY structures. Crystal structures of full-length DmCRY are available^19^,^20^,^21^, but not of HsCRY1/2. However, structures of the photolyase homology region (PHR) including the FAD-binding domains of *Mus musculus* CRY (MmCRY) 1 and 2 have been published, and have 93.6% and 94.6% sequence identity with HsCRY1 and 2, respectively^19^,^28^. Moreover, these sequence differences are peripheral, located far away from the putative FAD-binding pocket, and the differing residues are of similar type (Fig. S9a,b). We are therefore confident that the MmCRY1/2 structures are excellent surrogates for HsCRY1/2, and have analysed the MmCRY2 structure as an example of Type II animal CRY. In the MTD simulations, conformational sampling was performed along two distances from the centre-of-mass of the FAD-binding pocket: *i*) to the centre-of-mass of the FAD isoalloxazine; *ii*) to the centre-of-mass of the FAD adenine (Fig. S10). In DmCRY, FAD populates two major configurations, where the adenine group flips from the U-shape to an elongated configuration (D2 and D1, respectively, in Fig. 2a,c). Overall, however, the cofactor remains firmly bound to the protein. By contrast, four configurations are evident for MmCRY2, which successively illustrate how FAD leaves the protein pocket (M1 to M4; Fig. 2b,d); in the minimum energy conformation of MmCRY2 the isoalloxazine is *ca*. 12 Å further from the binding pocket than in DmCRY. These simulations demonstrate a major difference in the FAD binding affinity of DmCRY and MmCRY2. Notably, MTD also indicate that the initial FAD configuration for MmCRY2 (M0; Fig. 2b,d), corresponding to that observed in its crystal structure^28^, is not a stable minimum on the potential energy surface. This questions the relevance of this mode of FAD-binding – which was achieved by soaking crystals in FAD-containing buffer – to biological function.

### Specific amino acid positions are necessary for FAD-binding in cryptochromes

**Figure 3:**
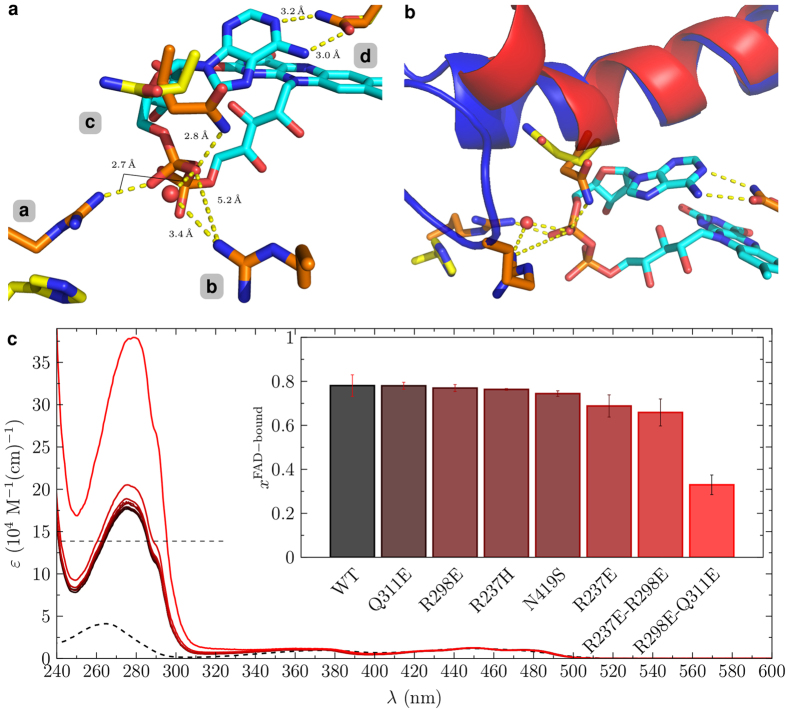
FAD binding site in typical Type I and Type II CRY and analysis by mutagenesis. (**a**) Structural alignment between the FAD-binding sites in DmCRY (orange, PDB code: 4GU5) and MmCRY2 (yellow, PDB code: 4I6G). Position (**a**) Arg237 (DmCRY), His242 (MmCRY2); (**b**) Arg298 (DmCRY); (**c**) Gln311 (DmCRY), Gln307 (MmCRY2); (**d**) Asn419 (DmCRY), Ser414 (MmCRY2). (**b**) Structural alignment of DmCRY and MmCRY2 with focus on the tertiary structure (blue – DmCRY; red – MmCRY2). (**c**) UV-visible absorption spectra of DmCRY wild type, variants, and free FAD (dashed line). The extinction coefficients are scaled to the absorbance maximum of FAD at 450 nm. Inset: FAD occupancy for each variant. The horizontal dashed line indicates the expected extinction coefficient at 280 nm of the holo-protein (100% FAD binding). (For details see Materials and Methods). Error bars shown in the inset are for three independent preparations of the cryptochrome proteins. Zoom into the FAD absorption bands in the spectral range from 300 to 550 nm can be found in Fig. S20.

**Figure 4:**
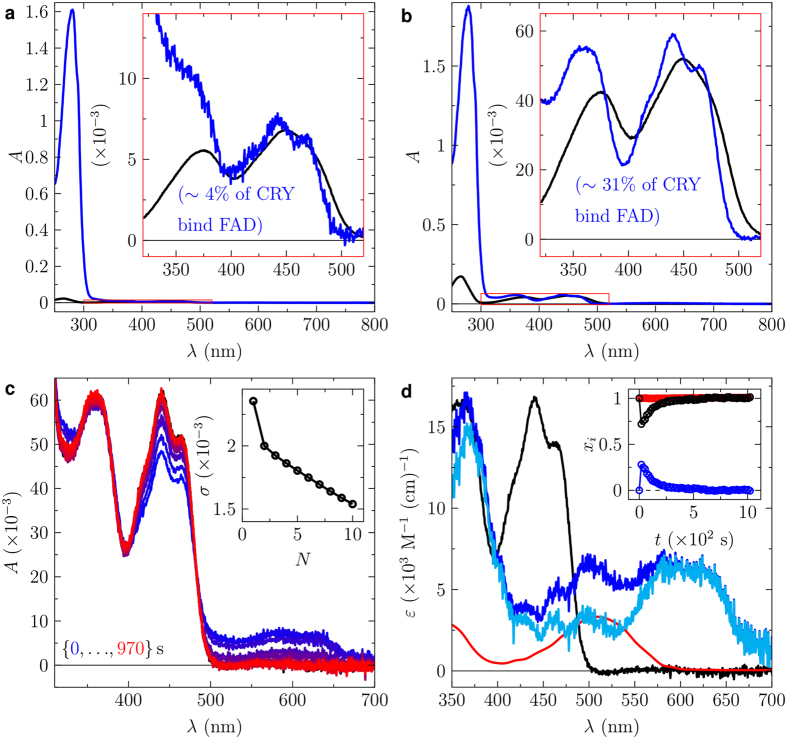
Recovering FAD-binding in the double mutant HsCRY2-H243R-S415N. (**a**) UV-visible absorption spectrum of HsCRY2-H243R-S415N (blue line) as purified from *E. coli* and unbound FAD (black line). (**b**) UV-visible absorption spectrum of HsCRY2-H243R-S415N (blue line) after incubation in excess FAD and removal of unbound FAD by spin-filtration. Unbound FAD (black line). (**c**) Sequence of UV-visible absorption spectra prior and post a 1s blue-light pulse (blue to red lines). The inset shows the singular value decomposition (SVD) based principle component analysis indicating two contributing species. (**d**) Extracted species spectra of bound oxidized FAD isoalloxazine (black), light-induced radical pair consisting of contributions of the neutral FAD semiquinone radical and the neutral tryptophan radical (blue line), neutral tryptophan radical (red line), and neutral FAD semiquinone radical (cyan line). The inset shows the mole fractions of bound oxidized FAD and light-induced radical pair. (For details see the main text and Materials and Methods).

**Figure 5:**
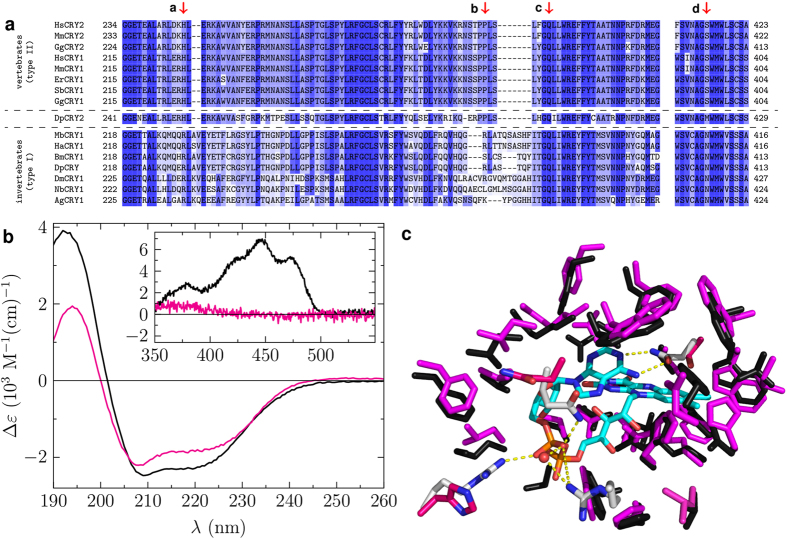
Sequence alignment of various Type I and Type II animal CRY and structural analysis of SbCRY1a. (**a**) Selected region of sequence alignment from Fig. S11 between eight Type I and eight Type II CRY. Colour coding: darker blue corresponds to higher sequence similarity. For abbreviations see caption of Fig. S11. Red arrows highlight the residues in position (**a**–**d**) in Fig. 3a. (**b**) CD spectra of DmCRY and SbCRY1a in the UV (main panel) and visible (inset). (**c**) Homology model of FAD binding to SbCRY1a (magenta) compared to DmCRY (black, PDB code: 4GU5). The specific amino acids at positions (**a**–**d**) from (panel a) are highlighted with pink carbon atoms for SbCRY1a and with white carbon atoms for DmCRY. The FAD is highlighted with cyan coloured carbon atoms.

There are important differences in the structure of the FAD-binding pocket in DmCRY compared to that of MmCRY2. Asn419 in DmCRY forms two H-bonds with the adenine of FAD^19^,^20^,^21^, stabilizing its distinctive U-shape (position **d** in Fig. 3a). The equivalent residue in MmCRY2, Ser414, is substantially shorter (position **d** in Fig. 3a)^28^. The FAD pyrophosphate group is stabilized in DmCRY through direct interactions with Arg237 and Gln311, and an interaction with Arg298 *via* a water molecule (positions **a**: Arg237, **b**: Arg298 and **c**: Gln311 in Fig. 3a). Replacement of Arg237 by His242 in MmCRY2 removes this stabilizing positive charge close to the pyrophosphate group. Although the glutamine is conserved, Gln307 in MmCRY2 points away from the pyrophosphate group owing to a kink in the α11 helix (position **c** in Fig. 3a,b). Arg298 is located in a loop region of DmCRY (amino acids 295 to 307) that acts as a lid of the FAD binding pocket (Fig. 3b). As noted previously, this loop is substantially truncated in MmCRY1^19^ and 2^28^, therefore does not act as a lid and introduces the kink in the α11 helix. We therefore made mutations to the positions identified in Fig. 3a (**a**: R237E/H, **b**: R298E, **c**: Q311E, and **d**: N419S) to disrupt FAD binding in DmCRY. The residues around the negatively-charged FAD pyrophosphate were replaced by glutamate – to retain a similar steric occupancy but disrupt the electrostatics – and Asn419 or Arg237 by the serine or histidine, respectively, as found in Mm/HsCRY. Apart from R237E, which shows a reduction of *ca*. 12%, single point mutations only had a minimal impact on FAD occupancy (Fig. 3c). We therefore hypothesized a cooperative effect of these interactions. Multiple double variants introducing a negative charge in order to disrupt the interaction to the FAD pyrophosphate produced soluble protein (R237E-R298E and R298E-Q311E) and resulted in further reductions in FAD occupancy by *ca*. 16 and 58%, respectively (Fig. 3c). Importantly, the FAD-binding pocket – especially in the region of the isoalloxazine moiety – seems not to be disturbed (see fine structural features in the FAD absorption bands in Fig. S20), which indicates that the overall protein conformation is intact. Furthermore, given that the FAD-binding pocket in DmCRY is more closed than that of MmCRY1/2 (≡HsCRY1/2), it is perhaps surprising that we were able to reduce FAD binding by more than 50% by a simple double mutation whilst leaving the backbone loop covering the FAD binding pocket intact. We also tried to replace the entire loop region in HsCRY2 (aa294–306) with the corresponding loop region in DmCRY (aa290–309). However, extensive purification trials indicated that this protein remained completely insoluble. We also performed a *vice versa* mutation study in HsCRY2. Since the glutamine at position **c** (see Figs 3a and 5a) is already in place and since HsCRY2 lacks the loop region present in DmCRY, we investigated the effect of replacing the histidine to arginine (HsCRY2-H243R) as well as the serine to asparagine (HsCRY2-S415N) at position **a** and **d**, respectively. These two variants were expressed and purified (see Fig. S1c,d), but FAD binding ability was not recovered (data not shown). The double mutation variant HsCRY2-H243R-S415N was expressed and purified (see Fig. S1e,f) with *ca*. 4% bound FAD (see Fig. 4a). Incubation in excess FAD and removal of free FAD afterwards by spin filtration resulted in an enhanced FAD binding of *ca*. 30% (see Fig. 4b). The double variant also showed photo-activity forming after a 1s blue-light pulse the radical pair of the neutral FAD semiquinone and the neutral tryptophan radicals (see Fig. 4c,d), which decayed within 250s. Thus, the combined computational and mutagenesis studies provide a molecular basis for the contrasting FAD-binding affinities evident in Figs 1 and 2 of Type I and II animal CRY.

### Structural features around FAD-binding pocket are consistent across Type I and Type II cryptochromes

To ascertain whether this differential binding of FAD is likely to be general across other Type I and Type II animal CRY, we performed a sequence alignment of eight of each type and mapped residue differences to structure (partial alignment, Fig. 5a; full alignment Fig. S11). Asn419 from DmCRY (position **d** in Fig. 3a) is highly conserved across all Type I CRY, whereas it is replaced by a serine or methionine in Type II. Arg237 in DmCRY (position **a** in Fig. 3a) is highly conserved in Type I and replaced by a histidine in Type II. The loop region around Arg298 (position **b** in Fig. 3a) that covers the FAD-binding pocket in DmCRY is present in all Type I, but like MmCRY2 is significantly truncated in Type II. Recall that this shorter backbone region also forces the α11 helix in MmCRY2 to kink such that the conserved glutamine residue does not interact with the pyrophosphate group (position **c** in Fig. 3b)^28^. These general features of Type I and II CRY indicate that their light-dependent and -independent functions, respectively, are owing to differential FAD binding.

The intracellular concentration of free FAD in human *HeLa* cells is estimated to be in the range of 100 nM to 3 μM^28^. The CRY concentration is suggested to be 10 nM to 1 μM, typical for signalling proteins^31^. The upper limit of FAD-bound Type II CRY2 *in vivo* is therefore expected to be between 4.3 to 7% based on either our determined *K*_D_ value of 68 μM or the reported *K*_D_ of 40 μM^28^. In case of FAD-bound Type II CRY1 the upper limit *in vivo* is only slightly higher at *ca*. 16% based on our determined *K*_D_value of 16 μM. This implies that under physiological conditions Type II CRY is predominantly cofactor-free. Further, this is emphasised by *in vitro* experiments in which high concentrations of FAD (500 μM) were required to see interruption of the interaction between MmCRY1 or MmCRY2 to either the small molecule KL001, or to the F-box protein FBXL3, respectively^28^,^32^.

We compared the MmCRY2/FBXL3 complex with the snapshots M0 to M4 from the metadynamics simulations from Fig. 2 (see Fig. S12). Interestingly, only the C-termini differ from each other. In the complex, the C-terminus of MmCRY2 is located away from the entrance of the FAD-binding pocket and remains open allowing entry of small molecules. In MmCRY2 alone, the C-terminus points towards the pocket entrance. Addition of FAD disrupts the complex, whereas FMN does not^26^ and this likely reflects additional binding energy associated with the additional phosphate and the adenine group of FAD.

### Type II cryptochromes are unlikely to be photoreceptors in animal magnetoreception

The sequence alignment in Fig. 5a includes examples of Type II CRY from birds (chicken, and the migratory European robin and garden warbler) where CRY is proposed to be the light-dependent magnetoreceptor^7^. In contrast to previous reports^25^ we were unable to isolate SbCRY1a (*Sylvia borin*; garden warbler; Fig. S1) with bound FAD, despite the protein being well-folded (Fig. 5b). During MTD simulations of a homology model of SbCRY1a with FAD incorporated (Fig. 5c), as with MmCRY2 and in contrast to DmCRY, the FAD ultimately leaves the protein pocket (Figs S13 and S14). The absorption spectrum of SbCRY1a in ref. 25 lacks the vibrational fine structure typical of FAD bound to CRY, which is consistent with either an absence of, weak, or non-specific FAD–CRY interactions. In contrast to this work, SbCRY1a showed a significant fine structure in the UV-visible absorption spectrum when excess SbCRY1a was added to 8 μM FAD giving good evidence of binding inside the protein pocket. We were also able to determine the *K*_D_ value to 97 μM demonstrating, again, a very low affinity towards FAD-binding which is lower than the values for HsCRY1 and 2 (see Fig. S15). Thus, the expected upper limit *in vivo* of SbCRY1a proteins binding FAD is only *ca*. 3%. In the case where FAD is bound to SbCRY1a, photo-induced reactivity between excited FAD isoalloxazine and aromatic amino acids of the protein backbone was observed resulting in the formation of the neutral FAD semiquinone radical and the counter neutral tryptophan radical with a lifetime of 340 ms (see Figs S16 and S17). The decay of the radical pair occurs within 60 s. The current chemical model of CRY as magnetoreceptor requires photoexcitation of FAD for generation of the magnetically sensitive radical pair^7^. This model is possible in Type I, DmCRY, which binds FAD and has been confirmed as necessary for the magnetic sense of *Drosophila melanogaster*^6^. However, our results throw significant doubt over such a photomagnetoreceptor role for Type II CRY in the avian (and similar vertebrate) compass, although a signalling role as a light-independent interaction protein cannot be ruled out. Since evidence exists that HsCRY2 can rescue the light-dependent magnetosensitivity in *Drosophila*^33^, our data suggest that CRY is unlikely to be the initial photoreceptor but is rather involved in the dark signalling pathway *via* protein interaction cascades.

## Discussion

The results presented herein are consistent with the respective circadian roles of Type I and Type II animal CRY. In *Drosophila melanogaster*, photochemically activated CRY – which correlates with flavin photoreduction^34^ – interacts with the clock protein, TIMELESS, as the first step in circadian photoentrainment^9^. Type II CRY (including mammals) on the other hand, regulate the central circadian feedback loop^15^ by inhibiting transcription of the *Per1* clock gene independently of light^17^. Although early evidence was initially considered consistent with a role for mammalian CRY as a circadian photoreceptor^35^,^36^, the discovery of melanopsin in combination with rod-cone photoreceptive systems have been accounted for all major mammalian circadian photoentrainment^37^. Although we showed for the first time unquestionable FAD-binding for Type II CRYs *in vitro*, as well as their photo-activity with the formation of the radical pair consisting of neutral FAD and neutral tryptophan radicals as the signalling state, the low FAD-binding affinity measured by dissociation constants in the ten to hundreds of μM range demonstrates that Type II CRYs predominantly do not bind FAD *in vivo* and should be seen as vestigial flavoproteins. Our results also pinpoint the structural features necessary to bind a photoactive chromophore which Type II CRYs lack excluding them from being a photoreceptor.

To date four different bird cryptochromes have been found (CRY1a, CRY1b, CRY2, and CRY4)^38^,^39^,^40^. However, it has been claimed that only one (CRY1a) of the four known bird cryptochromes can be found in all ultraviolet/violet cone cells in the retinas of both European robins and chickens^41^. Thus, CRY1a was proposed to be the potential candidate for photomagnetoreception. However, based on our findings CRY1a is unlikely to be a photomagnetoreceptor; its action is likely restricted to signal transduction in the dark *via* protein interaction cascades.

The CRY4 sequence information is available for the non-migrating chicken^42^ and other animal species, *e.g*. zebrafish^43^. For those species CRY4 shows flavin binding and light-dependent conformational changes similar to *Drosophila* CRY. It would be of interest to compare the CRY4 of migratory birds (e.g. the garden warbler) with our study. While the existence of CRY4 in the migratory garden warbler has only been shown^38^, neither the entire sequence, and thus nor any structural data, are yet available.

## Materials and Methods

### Expression and Purification of CRY

The construct design and the protein expression were adapted from ref. 44. In brief, DNA encoding the respective CRY protein (residues: DmCRY 1–539, HsCRY1 1–586, HsCRY2 1–593, SbCRY1a 1–620) was sub-cloned into the expression vector pRSET-A (Invitrogen) *via* XhoI and HindIII restriction sites leading to a 39 amino acid N-terminal extension of the proteins. In the case of SbCRY1a, the restriction sites BamHI and HindIII were used (36 aa extension). The sequence of the genes was confirmed by DNA sequencing (MWG Eurofins). The proteins were recombinantly expressed in *Escherichia coli* SoluBL21 (Amsbio) as described in ref. 44. The 6xHis-tagged proteins were purified by Ni-NTA (HisTrap FF, GE Healthcare) affinity chromatography in Tris buffer (pH = 7.5) containing 500 mM NaCl, 10 mM imidazole and 20% glycerol. Non-specifically bound proteins were washed away with binding buffer containing 30 mM imidazole followed by elution of the column-bound proteins with binding buffer containing 300 mM imidazole. The proteins were desalted on a CentriPure P100 desalting column (Generon) (50 mM HEPES, 50 mM NaCl and 10% glycerol at pH = 8.0 for DmCRY; pH = 9.0 for HsCRY1, and SbCRY1a) and were subjected to anion exchange chromatography. The proteins were purified using a linear gradient from 50 to 300 mM NaCl on the ResourceQ column (GE Healthcare). After desalting (same procedure and pH as described above unless otherwise stated), the proteins were further purified on a MonoQ 10/100GL column (GE Healthcare) using a linear gradient from 50 to 300 mM NaCl. HsCRY2 was purified by cation exchange chromatography on a ResourceS column (GE Healthcare) in HEPES buffer at pH = 7.0 followed by anion exchange chromatography on a MonoQ 10/100GL column at pH = 8.0, respectively, as described before. The proteins were desalted on a CentriPure P5 desalting column (Generon) with either a 50 mM HEPES buffer (pH = 8.0) containing 150 mM NaCl and 10% glycerol for UV-visible absorption measurements or 20 mM Na_2_HPO_4_, 50 mM NaCl and 10% glycerol buffer (pH = 8.0) for CD measurements. The homogeneity and purity of the isolated CRY proteins was verified by SDS-PAGE after each preparation using 12% polyacrylamide gels (Mini-Protean TGX Stain-FreeTM Gel with Precision Plus Protein™ Unstained Standards, BioRad) followed by either proteolysis with trypsin and mass spectrometry using MASCOT analysis^45^,^46^ or Western Blot analysis.

### Site-directed mutagenesis

Mutagenesis was performed by using the Q5 Site-Directed Mutagenesis Kit (New England BioLabs) and verified by sequencing (MWG Eurofins). The following primers (MWG Eurofins) were used to generate the mutant constructs: DmCRY-R237H forward 5′ gctggatgaaCATctgaaagttgaacag 3′, DmCRY-R237E forward 5′ gctggatgaaGAActgaaagttgaacagcatgcatttg 3′, DmCRY-R237H/E reverse 5′ agcagcagtgcctgggtt 3′; DmCRY-R298E forward 5′ tgcatgtgttGAAggtgttcagatgac 3′, DmCRY-R298E reverse 5′ cgcagctgaacgtttttaaac 3′; DmCRY-Q311E forward 5′ tatcaccggtGAActgatttggcg 3′, reverse DmCRY-Q311E 5′ tgtgcaccaccggtcatc 3′; DmCRY-N419S forward 5′ ttgtgcaggtAGCtggatgtgggttag 3′ reverse 5′ acgctccaatctgcatcc 3′; HsCRY2-H243R forward 5′ cctggataaaCGTctggaacgcaaagcg 3′, reverse 5′ cgcgccagcgcttcggtt 3′; HsCRY2-S415N forward 5′ gaacgcgggcAACtggatgtggc 3′, reverse 5′ acgctaaaatccgcatccagcag 3′. Variant proteins were expressed and purified as described for the wild type proteins.

### Western blot analysis

The proteins were transferred onto a 0.2 μm PVDF membrane for 10 min at 25 V and 1.3 A using the Transfer-Blot Turbo Transfer System (BioRad). For Western blot analysis the iBind Western System (Novex) was used. The membrane was incubated with the monoclonal mouse anti-His-Tag antibody (Novagen) and WesternSure HRP-conjugated goat anti-mouse IgG (Li-COR) for 2.5 h at room temperature. The detection was performed by incubating the membrane for 5 min in the WesternSure Premium Chemiluminescent Substrate (Li-COR) and scanning the blot using C-DiGit Blot Scanner (Li-COR).

### Stationary circular dichroism absorption spectroscopy

Stationary circular dichroism absorption spectra were recorded using an Applied Photophysics Chirascan CD-Spectrophotometer at 20 °C. In the UV range from 180 to 350 nm a pathlength of 100 μm and in the visible range from 350 to 600 nm a pathlength of 10 mm was used. The buffer background was subtracted from each spectrum.

### Stationary UV-visible absorption spectroscopy

Stationary UV-visible absorption spectra were recorded using an Agilent Cary 50 UV-Vis Spectrophotometer. A sample of DmCRY was illuminated using a pulsed, high-power light-emitting diode (LED; M455L3, Thorlabs) with *λ*_max_ = 455 nm. The 1s rectangular excitation pulse (20 mJ) was collimated using an anti-reflection-coated aspheric lens (Thorlabs), and delivered along the 2 mm pathlength of the cuvette orthogonal to the detection beam. After excitation an absorption spectrum was recorded directly afterwards. The sample volume was 120 μL with an optical density over 1 cm of 0.5 at the excitation wavelength. Thus, the entire sample volume was homogeneously illuminated, avoiding possible effects of diffusion during the recording of the spectra. The illumination produced the FAD radical anion due to electron transfer from electron donating amino acids, *e.g*., tryptophans, to the excited S_1_ state of the FAD. Since the conversion is not complete the obtained spectrum after a single blue-light pulse still includes contributions of the FAD in its oxidized form. The spectra of either the pure anionic FAD radical or the neutral FAD radical in the corresponding CRY proteins were generated by subtracting the spectrum of the oxidized FAD in the corresponding CRY protein from the spectrum after blue-light illumination until its characteristic fine structural features disappear and the spectrum resembles those of other published spectra for the individual radical species. In the case where the neutral FAD radical was formed, we observed for all Type II CRYs some deviations – especially in the range between 450 to 550 nm – that can be explained in good agreement to known spectra by the contributions of the tryptophan neutral radical. Thus, by subtracting a certain amount of the neutral tryptophan radical from the mixture spectrum it was possible to obtain a perfect match to the known flavin neutral radical spectra. As reference spectra we used: anionic FAD radical, *e.g*. refs 47 and 48; neutral FAD radical, *e.g*. refs 47 and 49; neutral tryptophan radical, *e.g*. ref. 50. The *K*_D_ values of FAD binding to each Type II CRY were estimated using a spectral method. Samples were prepared containing different concentrations of CRY apo-protein and a constant concentration (8 μM) of FAD. An UV-visible absorption spectrum was acquired for each concentration of apo-protein used (see inset of Figs S3a, S4a and S15a) giving the spectral evolution from the unbound FAD spectrum into the almost fully bound FAD spectrum with the typical vibrational fine structural features when bound to a protein pocket. This data matrix can be decomposed into its principle species spectra and corresponding mole fraction profiles (see Figs S3b, S4b and S14b). Here the mole fractions were determined by fitting the pure spectra of unbound and bound FAD to each single mixed spectrum. Since the bound FAD spectrum is *a priori* unknown, it is initially estimated by the last spectrum (highest concentration of apo-protein) in the spectral sequence. Each mixed spectrum also contains an absorptive contribution due to scattered light from the sample (especially at high concentrations of apo-protein), which was accounted for by a general scatter function of the following form:


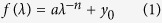


where *λ* is the wavelength, *y*_0_ accounts for the offset, *a* scales the curve, and *n* determines the curvature of the scatter function. *n* was fixed to −2 representing a reasonable curvature of the scatter contribution. Here the mole fraction profiles show a typical binding curve of the following chemical equilibrium:


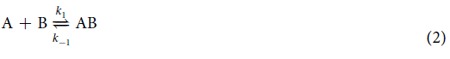


where,


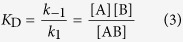


The amount of complex AB, *x*_AB_, is dependent on [A] and [B], the concentration of the two binding partners, and is given by:


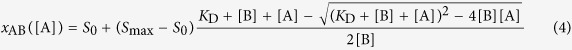


[A] is the independent variable representing the concentration of the apo-protein, [B] corresponds to the 8 μM fixed FAD concentration, *K*_D_ is the dissociation constant, and *S*_0_ and *S*_max_ represent the minimal and maximal limits of the binding curve, respectively. The mole fractions for unbound and in Type II CRY bound FAD were globally least squared fitted to a common *K*_D_ using the Levenberg-Marquardt algorithm. The first global fit was used to determine the contamination of the unbound FAD spectrum. Then the initial guess spectrum and the mole fractions were corrected correspondingly yielding the pure in Type II CRY bound FAD spectrum as well as mole fractions ranging from 0 to 1 as expected. The amount of bound FAD in DmCRY and the corresponding variants, *x*^FAD-bound^, was estimated by assuming that the extinction coefficients of FAD and the corresponding apo proteins are additive at 280 nm. The extinction coefficient at 280 nm of FAD, *ε*^FAD^ (280 nm), was determined in the used buffer system to 22869 M^−1^ (cm)^−1^. The extinction coefficient of the apo protein at 280 nm, *ε*^apo^ (280 nm), was estimated by the Edelhoch method^51^, but with the extinction coefficients for Trp and Tyr determined by Pace^52^, to 115850 M^−1^ (cm)^−1^. The concentration of FAD bound in each individual protein, *c*^FAD-bound^, was determined *via* the absorption at 450 nm and the corresponding determined extinction coefficient, *ε*^FAD^ (450 nm) = 12500 M^−1^ (cm)^−1^. Then, the amount of bound FAD in DmCRY and the corresponding variants, *x*^FAD-bound^, was calculated by the following equation:


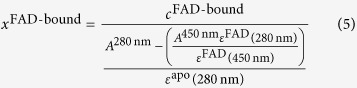


where *A*^280 nm^ is the absorption at 280 nm.

### Homology modelling and metadynamics simulations

The program modeller (version 9.11)^53^ was used to generate full-length homology model structures of HsCRY2, and SbCRY1a. As a basis for the homology model the available structures of DmCRY (pdb code: 4GU5)^20^, MmCRY1 (pdb code: 4K0R)^19^ and MmCRY2 from *Mus musculus* (pdb code: 4I6G)^28^ were used. The homology modelling process included an initial sequence alignment based on the program ClustalX (version 1.83)^54^,^55^ with the following parameters: Gap Opening: 10; Gap Extension: 0.2; Delay Divergent Sequence: 30%; Use Negative Matrix: yes; Protein Weight Matrix: BLOSUM series).

Molecular dynamics (MD) simulations were performed with the Gromacs package^56^ and the Gromos53a6 force field^57^, a solvation box of minimum 1 nm around the protein and periodic boundary conditions. The initial system set-up was performed as follows: after energy minimization the system was thermalized 300 K for 100 ps using *NVT* dynamics, and the pressure was then equilibrated for 100 ps using *NPT* dynamics; the protein and the flavin adenine dinucleotide (FAD) were constrained during these steps. All constraints and pressure couplings were then switched off and the system relaxed using *NVT* at 250 K, 280 K, 290 K and 300 K for 1 ns each, before the production MD runs which were performed at 300 K. Well-tempered metadynamics (MTD) simulations (based on^30^) were performed using the PLUMED library^58^, using structures after 10 ns of conventional MD (black trace in Fig. S18). Metadynamics is a method of enhanced sampling that calculates the free energy surface along predefined Collective Variables (CVs) by applying Gaussian bias potentials along the CVs as these are sampled during the simulation. Well-tempered MTD additionally uses a bias factor to decrease the magnitude of the bias potential at CV values that are heavily sampled, which ensures rapid convergence. In this case two CVs were applied to the system – the distance between the isoalloxazine and binding pocket residues and the distance between the adenine and the same residues (Fig. S10). Five 100 ns MTD simulations were carried for each protein, using a different seed for the initial velocity generation, with bias potentials of 0.3 Å width, 4.0 kJ mol^−1^ height, and a bias factor ranging from 10 to 15 (15 for the first two, 12.5 for the third and 10 for the last two in Fig. S13). The results shown in Fig. 2 in the main text are the average free energy surfaces; the individual surfaces for each protein are shown in Fig. S13, and the values of the CVs during each simulation are shown in Fig. S14. Additionally, one simulation for MmCRY2 and DmCRY was performed for 300 ns, shown in Fig. S19.

## Additional Information

**How to cite this article**: Kutta, R. J. *et al*. Vertebrate Cryptochromes are Vestigial Flavoproteins. *Sci. Rep.* **7**, 44906; doi: 10.1038/srep44906 (2017).

**Publisher's note:** Springer Nature remains neutral with regard to jurisdictional claims in published maps and institutional affiliations.

## Supplementary Material

Supplementary Information
