# Supplementary material for: Vertebrate Cryptochromes are Vestigial Flavoproteins
Source: Sci Rep. 2017 Mar 20;7:44906. doi: 10.1038/srep44906 (PMC5357904; doi:10.1038/srep44906)
Supplement: Supplementary Information [file srep44906-s1.pdf]

Supplementary Information

Vertebrate Cryptochromes are Vestigial Flavoproteins

Roger J. Kutta,[1,2*] Nataliya Archipowa,[1] Linus O. Johannissen,[1] Alex R. Jones,[1,2*] and Nigel S. Scrutton[1*]

1. Manchester Institute of Biotechnology (MIB) and School of Chemistry, The University of Manchester, 131 Princess Street, Manchester, M1 7DN, UK.

2. Photon Science Institute and School of Chemistry, The University of Manchester, Alan Turing Building, Oxford Road, Manchester, M13 9PL, UK.

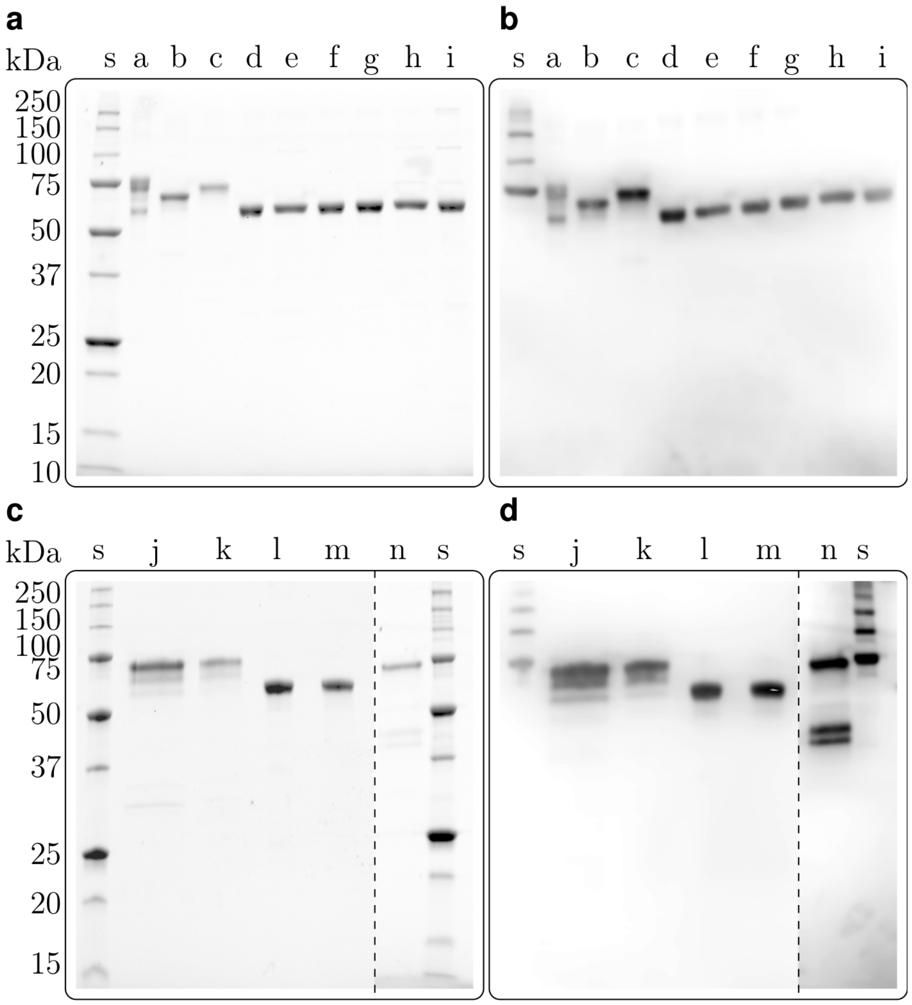

Figure S1. SDS-PAGE and Western blot analysis of purified CRY proteins. a and **c**: SDS-PAGE of Marker (s; Precision Plus Protein™ Unstained Standards, BioRad), SbCRY1a (a), HsCRY1 (b), HsCRY2 (c), DmCRY (d), DmCRY-R237E (e), DmCRY-R298E (f), DmCRY-Q311E (g), DmCRY-R237E-R298E (h), DmCRY-R298E-Q311E (i), HsCRY2-H243R (j), HsCRY2-S415N (k), DmCRY-R237H (l), DmCRY-N419S (m), and HsCRY2-H243R-S415N (n). **b** and **d**: Corresponding Western blot of **a** and **c**. The multiple bands in lanes (a), (j) and (k) indicate some minor degradation of the protein. Selected proteins (the first four marker bands) in the commercial strep-tag marker sample also cross-react with anti-His antibodies used in panels **b** and **d**. The dashed lines in panels **c** and **d** indicate the junctions between two individual gels/blots.

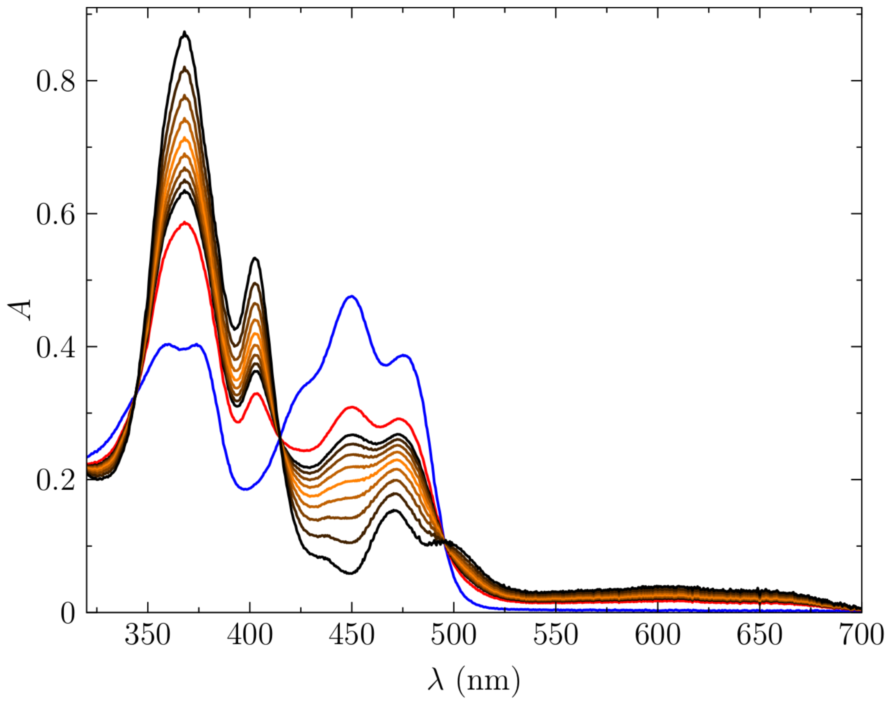

Figure S2. Determination of the pure FAD radical anion spectrum in DmCRY *via in silico* titration. The absorption spectra of DmCRY were recorded before (blue line) and directly after illumination with a 1 s blue-light pulse (red line). The spectrum of the pure FAD radical anion was generated by subtracting the spectrum of the oxidized FAD from the spectrum after blue-light illumination until the signatures of the spectrum of the oxidized FAD disappeared. The spectral sequence from black to orange to black corresponds to contributions of the oxidized spectrum of 20% to 60% in 5% steps, respectively.

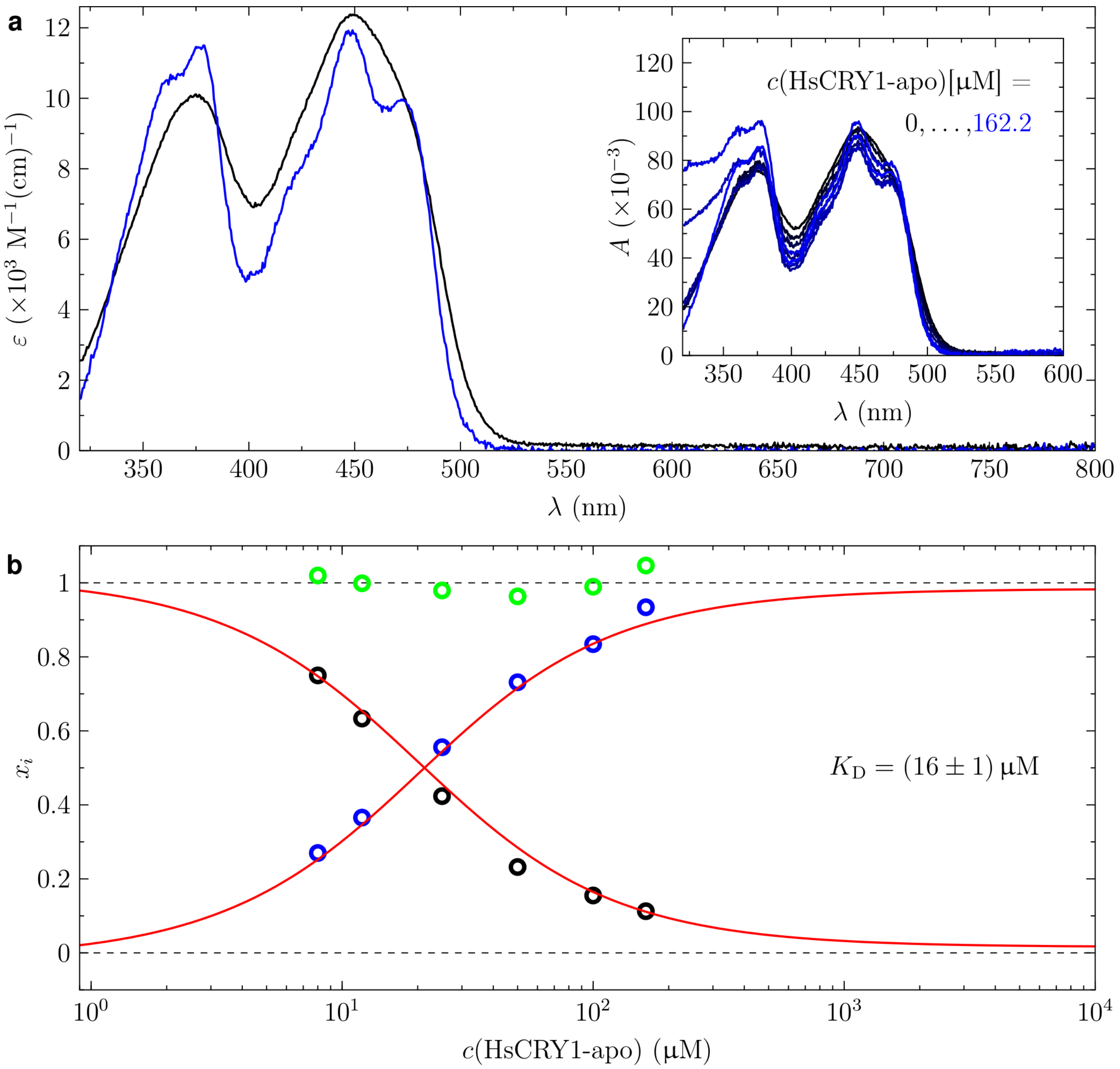

Figure S3. Determination of the FAD-binding affinity in HsCRY1. a: pure UV-visible absorption spectra of unbound FAD (black) and bound in HsCRY1 (blue) extracted from the raw data (inset in panel **a**: raw data minus fitted scatter contribution) as described in the Materials and Methods section of the main text. **b**: mole fractions of unbound FAD (black open circles) and bound in HsCRY1 (blue open circles) as determined from fitting the pure spectra from panel **a** to each spectrum of the inset in panel **a** correlated with the HsCRY1-apo concentration. The green open circles represent the sum of the mole fractions. The red lines represent the global fits of the binding equation 4 to the data.

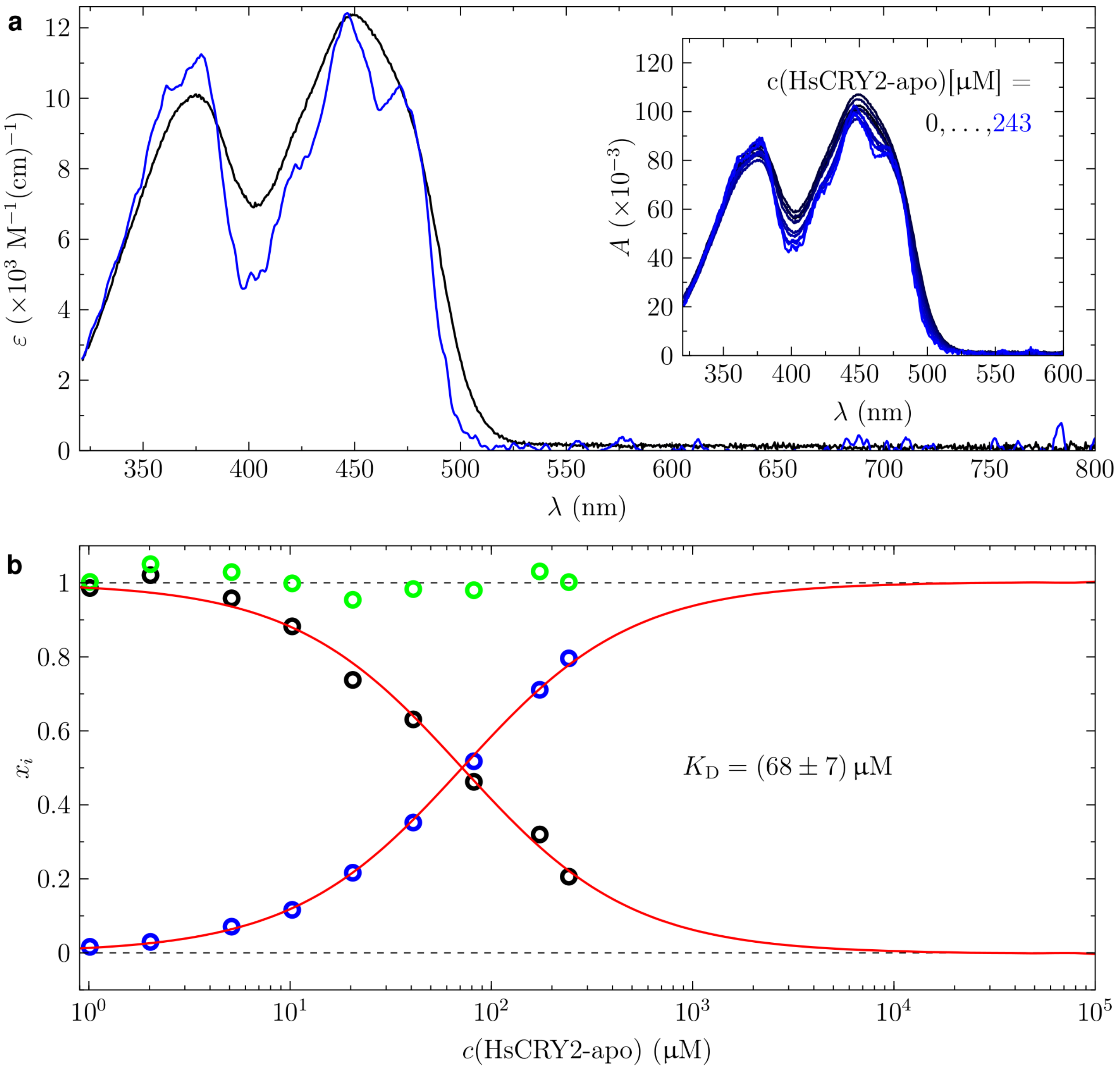

Figure S4. Determination of the FAD-binding affinity in HsCRY2. a: pure UV-visible absorption spectra of unbound FAD (black) and bound in HsCRY2 (blue) extracted from the raw data (inset in panel **a**: raw data minus fitted scatter contribution) as described in the Materials and Methods section of the main text. **b**: mole fractions of unbound FAD (black open circles) and bound in HsCRY2 (blue open circles) as determined from fitting the pure spectra from panel **a** to each spectrum of the inset in panel **a** correlated with the HsCRY2-apo concentration. The green open circles represent the sum of the mole fractions. The red lines represent the global fits of the binding equation 4 to the data.

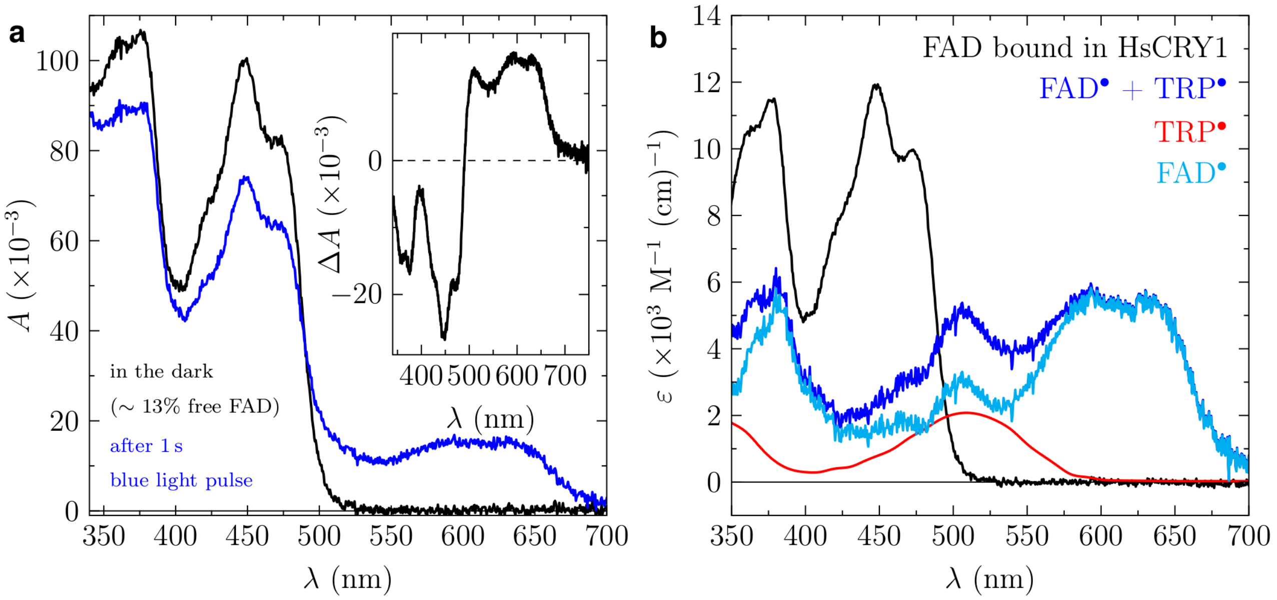

Figure S5. Determination of the pure neutral FAD radical spectrum and the counter neutral tryptophan radical in HsCRY1 *via in silico* titration. Absorption spectra of a sample containing 8 µM FAD and *ca*. 100 µM HsCRY1 before (black line in panel **a**) and directly after illumination with a 1 s blue-light pulse (blue line in panel **a**). Since this sample still contained *ca*. 13% of unbound FAD, the difference spectrum (inset in panel **a**) subtracts the contribution of unbound FAD. Adding the pure spectrum of fully bound FAD to HsCRY1 from Figure S3**a** to the difference spectrum in such a way that its characteristic fine structural features disappear, results in a spectrum (blue line in panel **b**) with a high similarity to known spectra for the neutral flavin semiquinone radical, *e.g.* shown in[1,2]. However, some deviations can still be seen especially in the range between 450 to 550 nm. These deviations can be explained by the contributions of the neutral tryptophan radical (red line extracted from[3]). By subtracting a certain amount of the neutral tryptophan radical from the mixture spectrum it is possible to obtain a perfect match to the known flavin neutral radical spectrum (cyan line). (A detailed time-resolved analysis of the mechanism including branching ratios, the photoproduct, and a discussion about the proton donor is beyond the scope of this work and will be presented elsewhere.)

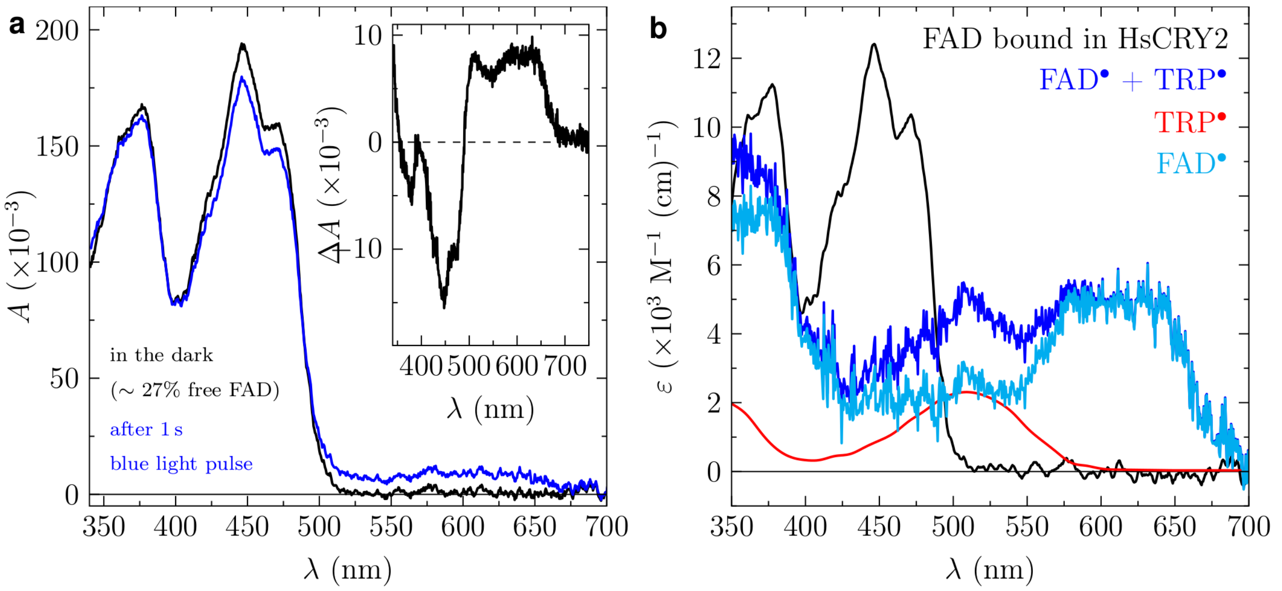

Figure S6. Determination of the pure neutral FAD radical spectrum and the counter neutral tryptophan radical in HsCRY2 *via in silico* titration. Absorption spectra of a sample containing 15.4 µM FAD and *ca.* 317 µM HsCRY2 before (black line in panel **a**) and directly after illumination with a 1 s blue-light pulse (blue line in panel **a**). Since this sample still contained *ca.* 27% of unbound FAD, the difference spectrum (inset in panel **a**) subtracts the contribution of unbound FAD. Adding the pure spectrum of fully bound FAD to HsCRY2 from Figure S4**a** to the difference spectrum in such a way that its characteristic fine structural features disappear, results in a spectrum (blue line in panel **b**) with a high similarity to known spectra for the neutral flavin semiquinone radical, *e.g.* shown in[1,2]. However, some deviations can still be seen especially in the range between 450 to 550 nm. These deviations can be explained by the contributions of the neutral tryptophan radical (red line extracted from[3]). By subtracting a certain amount of the neutral tryptophan radical from the mixture spectrum it is possible to obtain a perfect match to the known flavin neutral radical spectrum (cyan line). (A detailed time-resolved analysis of the mechanism including branching ratios, the photoproduct, and a discussion about the proton donor is beyond the scope of this work and will be presented elsewhere.)

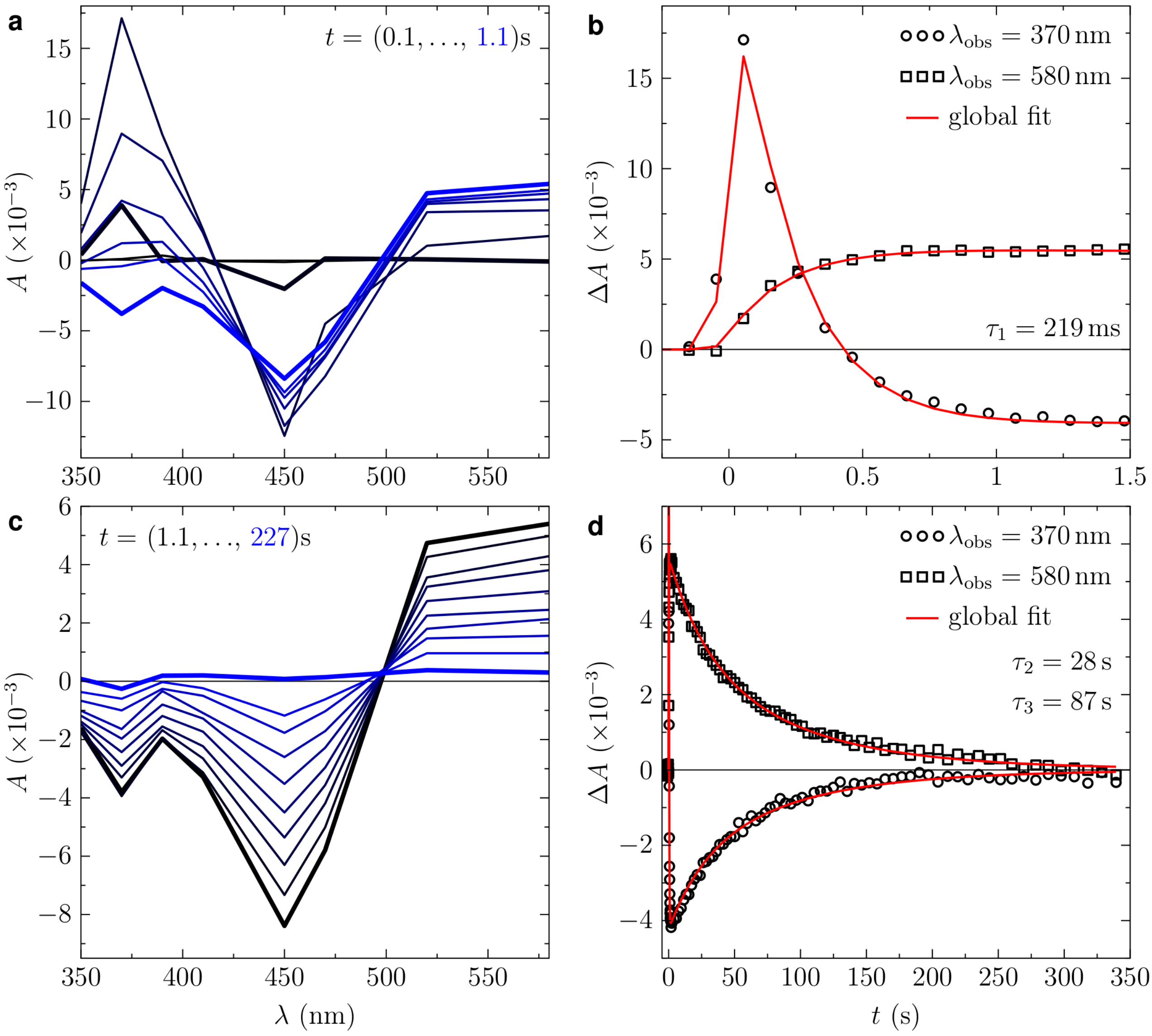

Figure S7. Photo-induced formation and decay of the neutral FAD semiquinone radical bound in HsCRY1. UV-visible absorption changes at single wavelengths in the range from 350 to 580 nm induced by single 100 ms blue-light pulse were recorded for a sample containing 8 μM FAD and *ca.* 100 μM HsCRY1. The spectrally (**a** and **c**) and temporally (**b** and **d**) resolved UV-visible absorption changes up to 1 s (**a** and **b**) and 350 s (**c** and **d**) are shown at two selected wavelength representative for the main features of the anionic (370 nm) and neutral (580 nm) FAD semiquinone radicals. The red lines represent the global fit of two exponential functions to the entire data matrix with the lifetimes of 219 ms, 28 s and 87 s. (A detailed time-resolved analysis of the mechanism including branching ratios, the photoproduct, and a discussion about the proton donor is beyond the scope of this work and will be presented elsewhere.)

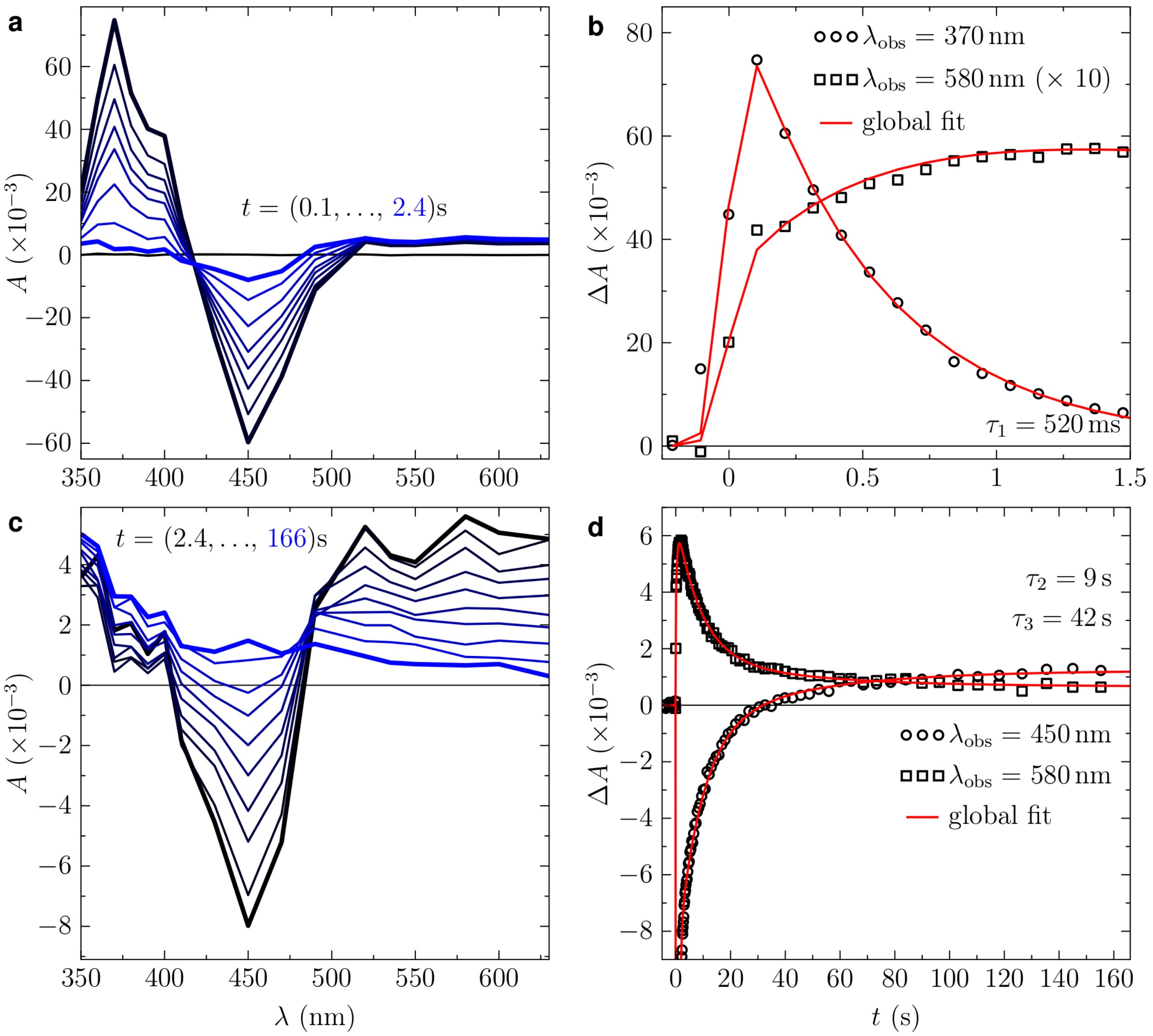

Figure S8. Photo-induced formation and decay of the neutral FAD semiquinone radical bound in HsCRY2. UV-visible absorption changes at single wavelengths in the range from 350 to 580 nm induced by single 100 ms blue-light pulse were recorded for a sample containing 15.4 µM FAD and *ca.* 317 µM HsCRY2. The spectrally (**a** and **c**) and temporally (**b** and **d**) resolved UV-visible absorption changes up to 1 s (**a** and **b**) and 350 s (**c** and **d**) are shown at three selected wavelength representative for the main features of the anionic (370 nm), the neutral (580 nm) FAD semiquinone radicals, and the oxidized FAD. The red lines represent the global fit of two exponential functions to the entire data matrix with the lifetimes of 520 ms, 9 s, and 42 s. (A detailed time-resolved analysis of the mechanism including branching ratios, the photoproduct, and a discussion about the proton donor is beyond the scope of this work and will be presented elsewhere.)

a

```
HsCRY2 1     MAATVATAAAVAPAPAPGTDSASSVHWFRKGLRLHDNPALLAAVRGARCVRCVYILDPWFAASSSVGINRWRFLLQSLEDLDTSL 85
MmCRY2 1     -MAAAAVVAATVPAQSMGADGASSVHWFRKGLRLHDNPALLAAVRGARCVRCVYILDPWFAASSSVGINRWRFLLQSLEDLDTSL 84

HsCRY2 86   RKLNSRLFVVRGQPADVFPRLFKEWGVTRLTFEYDSEPFGKERDAAIMKMAKEAGVEVVTENSHTLYDLDRIIELNGQKPPLTYK 170
MmCRY2 85   RKLNSRLFVVRGQPADVFPRLFKEWGVTRLTFEYDSEPFGKERDAAIMKMAKEAGVEVVTENSHTLYDLDRIIELNGQKPPLTYK 169

HsCRY2 171 RFQAIISRMELPKKPVGLVTSQQMESCRAEIQENHDETYGVPSLEELGFPTEGLGPAVWQGGETEALARLDKHLERKAWVANYER 255
MmCRY2 170 RFQALISRMELPKKPAVAVSSQQMESCRAEIQENHDDTYGVPSLEELGFPTEGLGPAVWQGGETEALARLDKHLERKAWVANYER 254

HsCRY2 256 PRMNANSLLASPTGLSPYLRFGCLSCRLFYYRLWDLYKKVKRNSTPPLSLFGQLLWREFFYTAATNNPRFDRMEGNPICIQIPWD 340
MmCRY2 255 PRMNANSLLASPTGLSPYLRFGCLSCRLFYYRLWDLYKKVKRNSTPPLSLFGQLLWREFFYTAATNNPRFDRMEGNPICIQIPWD 339

HsCRY2 341 RNPEALAKWAEGKTGFPWIDAIMTQLRQEGWIHHLARHAVACFLTRGDLWVSWESGVRVFDELLLDADFSVNAGSWMWLSCSAFF 425
MmCRY2 340 RNPEALAKWAEGKTGFPWIDAIMTQLRQEGWIHHLARHAVACFLTRGDLWVSWESGVRVFDELLLDADFSVNAGSWMWLSCSAFF 424

HsCRY2 426 QQFFHCYCPVGFGRRTDPSGDYIRRYLPKLKAFPSRYIYEPWNAPESIQKAAKCIIGVDYPRPIVNHAETSRLNIERMKQIYQQL 510
MmCRY2 425 QQFFHCYCPVGFGRRTDPSGDYIRRYLPKLKGFPSRYIYEPWNAPESVQKAAKCIIGVDYPRPIVNHAETSRLNIERMKQIYQQL 509

HsCRY2 511 SRYRGLCLLASVPSCVEDLSHPVAEPSSSQAGSMSSAGPRPLPSGPASPKRKLEAAEEPPGEELSKRARVAELPTPELPSKDA   593
MmCRY2 510 SRYRGLCLLASVPSCVEDLSHPVAEPGSSQAGSISNTGPRALSSGPASPKRKLEAAEEPPGEELTKRARVTEMPTQEPASKDS   592
```

b

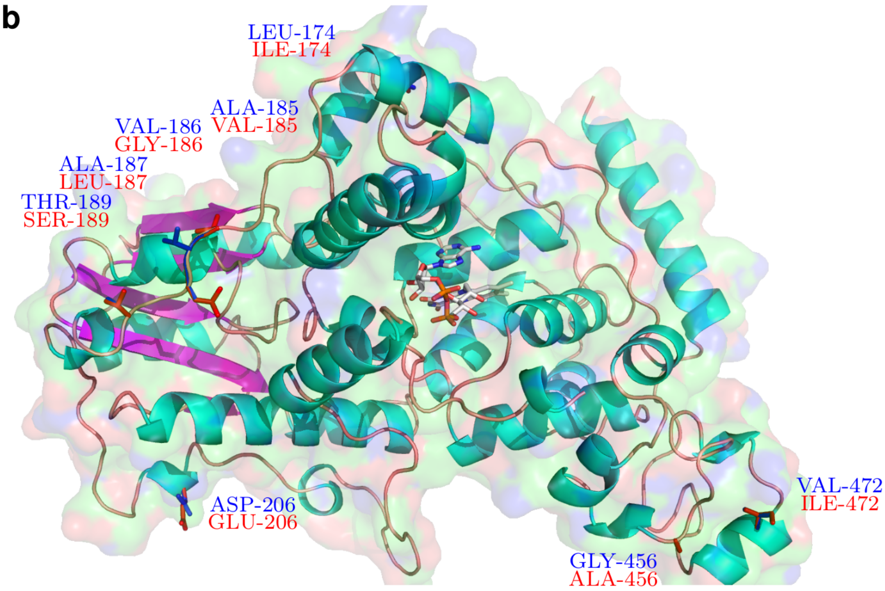

Figure S9. Structure and sequence alignment between HsCRY2 and MmCRY2. a: Sequence alignment. The differing amino acids between HsCRY2 and MmCRY2 are highlighted with a red background. **b**: Structural alignment. HsCRY2 is a homology model based on the template structure from MmCRY2 (yellow, PDB code: 4I6G); all secondary elements are therefore identical and only the resolved amino acids could be modelled. All differing amino acids as well as the FAD are shown as stick representation.

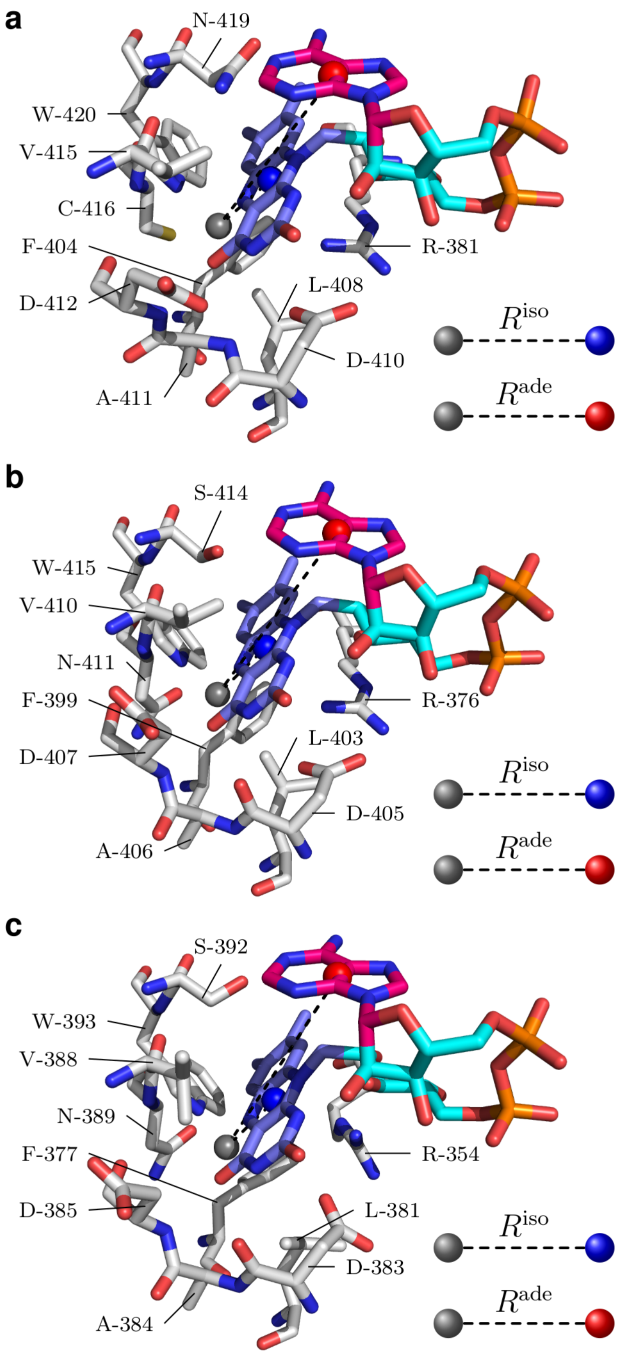

Figure S10. Definition of collective variables (CVs) used in metadynamics simulations. R^{iso} is defined as the centre-of-mass from the isoalloxazine (blue sphere) to the centre-of-mass of amino acid residues (gray sphere) and R^{ade} as the centre-of-mass from the adenine moiety (red sphere) to the same centre-of-mass of amino acid residues (gray spheres) involved in FAD-binding as represented. **a**: DmCRY. **b**: MmCRY2. **c**: SbCRY1a.

```
HsCRY2/1-593     1   -------MAATVATAAAVAPAPAPGTDSASSVHWFRKGLRLHDNPALLAAVRG--------ARCVRCVYILDPWFAASSSVGINRWRFLLQSLEDLDTSL   85
MmCRY2/1-592     1   --------MAAAAVVAATVPAQSMGADGASSVHWFRKGLRLHDNPALLAAVRG--------ARCVRCVYILDPWFAASSSVGINRWRFLLQSLEDLDTSL   84
GgCRY2/1-582     1   -------------MAAAASPPRG----FCRSVHWFRRGLRLHDNPALQAALRG--------AASLRCIYILDPWFAASSAVGINRWRFLLQSLEDLDNSL   75
HsCRY1/1-586     1   --------------------------MGVNAVHWFRKGLRLHDNPALKECIQG--------ADTIRCVYILDPWFAGSSNVGINRWRFLLQCLEDLDANL   66
MmCRY1/1-606     1   --------------------------MGVNAVHWFRKGLRLHDNPALKECIQG--------ADTIRCVYILDPWFAGSSNVGINRWRFLLQCLEDLDANL   66
ErCRY1/1-620     1   --------------------------MGVNAVHWFRKGLRLHDNPALRECIRG--------ADTVRCVYILDPWFAGSSNVGINRWRFLLQCLEDLDANL   66
SbCRY1/1-620     1   --------------------------MGVNAVHWFRKGLRLHDNPALRECIQG--------ADTVRCVYILDPWFAGSSNVGINRWRFLLQCLEDLDANL   66
GgCRY1/1-621     1   --------------------------MGVNAVHWFRKGLRLHDNPALRECIRG--------ADTVRCVYILDPWFAGSSNVGINRWRFLLQCLEDLDANL   66
- - - - - - - - - - - - - - - - - - - - - - - - - - - - - - - - - - - - - - - - - - - - - - - - - - - - - - - - - - - -
DpCRY2/1-742     1   MSVAETLPLRARSPTAQKSSQPAGVPKEKHTVHWFRKGLRLHDNPALREGLVD--------ATTFRCVFIIDPWFASSSNVGINKWRFLLQCLEDLDKNL   92
MbCRY1/1-548     1   --------------------------MLGGSVLWFRHGLRLHDNPSLHSALE-------EKGFPFFPVFIFDGETAGTKVVGYNRMRYLLEALEDLDNQL   67
HaCRY1/1-528     1   --------------------------MLGGSVLWFRHGLRLHDNPSLHSALE-------EKGFPFFPIFIFDGETAGTKLVGYNRMRYLLEALDDLDSQF   67
BmCRY1/1-536     1   --------------------------MLGGSVLWFRHGLRLHDNPSLHSALE-------ETSGPFFPIFIFDGETAGTKVVGYNRMRYLLEALDDLDKQF   67
DpCRY/1-534      1   --------------------------MLGGNVIWFRHGLRLHDNPSLHSALE-------DASSPFFPIFIFDGETAGTKMVGYNRMRYLLEALNDLDQQF   67
DmCRY1/1-542     1   ------------------------MATRGANVIWFRHGLRLHDNPALLAALAD-----KDQGIALIPVFIFDGESAGTKNVGYNRMRFLLDSLQDIDDQL   71
NbCRY1/1-542     1   ---------------------------MSLNIIWFRHGLRLHDNPALLDAISD-----KDEGIELLPIFIFDGESAGTQSVGYNRLKFLLDSLEDINNQL   68
AgCRY1/1-545     1   --------------------------MTINNILWFRHGLRLHDNPSLLEALKSDCVNQSSEAVKLFPIFIFDGESAGTRIVGYNRMKFLLESLADLDRQF   74

HsCRY2/1-593    86   RKLN---SRLFVVRGQPADVFPRLFKEWGVTRLTFEYDSEPFGKERDAAIMKMAKEAGVEVVTENSHTLYDLDRIIELNGQKPPLTYKRFQAIISRMELP   182
MmCRY2/1-592    85   RKLN---SRLFVVRGQPADVFPRLFKEWGVTRLTFEYDSEPFGKERDAAIMKMAKEAGVEVVTENSHTLYDLDRIIELNGQKPPLTYKRFQALISRMELP   181
GgCRY2/1-582    76   RKLN---SRLFVVRGQPTDVFPRLFKEWGVTRLAFEYDSEPFGKERDAAIIKLAKEAGVEVVIENSHTLYNLDRIIELNGNKPPLTYKRFQAIISRMELP   172
HsCRY1/1-586    67   RKLN---SRLFVIRGQPADVFPRLFKEWNITKLSIEYDSEPFGKERDAAIKKLATEAGVEVIVRISHTLYDLDKIIELNGGQPPLTYKRFQTLISKMEPL   163
MmCRY1/1-606    67   RKLN---SRLFVIRGQPADVFPRLFKEWNITKLSIEYDSEPFGKERDAAIKKLATEAGVEVIVRISHTLYDLDKIIELNGGQPPLTYKRFQTLVSKMEPL   163
ErCRY1/1-620    67   RKLN---SRLFVIRGQPADVFPRLFKEWNIAKLSIEYDSEPFGKERDAAIKKLASEAGVEVIVRISHTLYDLDKIIELNGGQPPLTYKRFQTLISRMEPL   163
SbCRY1/1-620    67   RKLN---SRLFVIRGQPADVFPRLFKEWNIAKLSIEYDSEPFGKERDAAIKKLASEAGVEVIVRISHTLYDLDKIIELNGGQPPLTYKRFQTLISRMEPL   163
GgCRY1/1-621    67   RKLN---SRLFVIRGQPADVFPRLFKEWSIAKLSIEYDSEPFGKERDAAIKKLASEAGVEVIVRISHTLYDLDKIIELNGGQPPLTYKRFQTLISRMEPL   163
- - - - - - - - - - - - - - - - - - - - - - - - - - - - - - - - - - - - - - - - - - - - - - - - - - - - - - - - - - - -
DpCRY2/1-742    93   RKLN---SRLFVVRGQPADALPKLFREWGTTALTFEEDPEPYGRVRDHNIMTKCREVGIQVTSRVSHTLYKLDDIIEKNGGKAPLTYHQFQALIASMPPP   189
MbCRY1/1-548    68   KKHG---GRLIMIKGKPNVVFRRLWEEFGIRRLCFEQDCEPVWRARDDSVKSACKEIGVVCKEHVSHTLWEPDTVIKANGGIPPLTYQMFLHTVATIGDP   164
HaCRY1/1-528    68   KKFG---GRLIMLKGKPNVVFRRLWEEFGIRKLCFEQDCEPVWRARDDSVKSACKEIGVVCREHVSHTLWEPETVIKANGGIPPLTYQMFLHTVATIGDP   164
BmCRY1/1-536    68   KKYG---GRLLLVKGKPSAVFRRLWEEFGIRKLCFEQDCEPVWRPRDESVKTACREIGVTCREHVSHTLWEPDTVIKANGGIPPLTYQMFLHTVATIGDP   164
DpCRY/1-534     68   RKYG---GKLLMIKGRPDLIFRRLWEEFGIRTLCFEQDCEPIWRPRDASVRALCRDIGVSCREHVAHTLWNPDTVIKANGGIPPLTYQMFLHTVEIIGNP   164
DmCRY1/1-542    72   QAATDGRGRLLVFEGEPAYIFRRLHEQVRLHRICIEQDCEPIWNERDESIRSLCRELNIDFVEKVSHTLWDPQLVIETNGGIPPLTYQMFLHTVQIIGLP   171
NbCRY1/1-542    69   RSVSLSLGRLYIMKGNPVQIFRSLHEQRGIKKLCFEQDCEPIWNRRDNAVKNLCHDLGITCIERISHTLWDPKKVINTNGGIPPLTYQMFLHTVQIIGLP   168
AgCRY1/1-545    75   RDLG---GQLLVFRGDSVTVLRRLFEELNIKKLCYEQDCEPIWKERDDAVAKLCRTMDVRCVENVSHTLWNPIEVIQTNGDIPPLTYQMFLHTVNIIGDP   171

HsCRY2/1-593   183   KKPVGLVTSQQMESCRAEIQENHDETYG---------------VPSLEELGFPTE---GLGPAVWQGGETEALARLDKHL--ERKAWVANYERPRMNANS   262
MmCRY2/1-592   182   KKPAVAVSSQQMESCRAEIQENHDDTYG---------------VPSLEELGFPTE---GLGPAVWQGGETEALARLDKHL--ERKAWVANYERPRMNANS   261
GgCRY2/1-582   173   KKPVSSIVSQQMETCKVDIQENHDDVYG---------------VPSLEELGFPTD---GLAPAVWQGGETEALARLDKHL--ERKAWVANYERPRMNANS   252
HsCRY1/1-586   164   EIPVETITSEVIEKCTTPLSDDHDEKYG---------------VPSLEELGFDTD---GLSSAVWPGGETEALTRLERHL--ERKAWVANFERPRMNANS   243
MmCRY1/1-606   164   EMPADTITSDVIGKCMTPLSDDHDEKYG---------------VPSLEELGFDTD---GLSSAVWPGGETEALTRLERHL--ERKAWVANFERPRMNANS   243
ErCRY1/1-620   164   EMPVETITPEVMKKCTTPVFDDHDEKYG---------------VPSLEELGFDTD---GLPSAVWPGGETEALTRLERHL--ERKASVANFERPRMNANS   243
SbCRY1/1-620   164   EMPVETITPEVMKKCTTPVSDDHDEKYG---------------VPSLEELGFDTD---GLPSAVWPGGETEALTRLERHL--ERKAWVANFERPRMNANS   243
GgCRY1/1-621   164   EMPVETITPEVMQKCTTPVSDDHDEKYG---------------VPSLEELGFDTD---GLPSAVWPGGETEALTRLERHL--ERKAWVANFERPRMNANS   243
- - - - - - - - - - - - - - - - - - - - - - - - - - - - - - - - - - - - - - - - - - - - - - - - - - - - - - - - - - - -
DpCRY2/1-742   190   PSAEPTISLETLNRAVTPISDNHDERFG---------------VPTLEELGFDTE---GLKPPIWIGGENEALLRLERHL--ERKAWVASFGRPKMTPES   269
MbCRY1/1-548   165   PRPVSDI-------------DFTGVKFGSLPESFYHEFTVYDKTPKPEDLGVFLE-NEDIRMIRWVGGETTALKQMQQRLAVEYETFLRGSYLPTHGNPD   250
HaCRY1/1-528   165   PRPVPNI-------------DFTGVKFGSLPECFYQEFTVYDKTPKPEDLGVFLE-NEDIRMIRWVGGETTALKQMQQRLSVEYETFLRGSYLPTHGNPD   250
BmCRY1/1-536   165   PRPVDNA-------------KLRGIKFGTLPLCFYEEFTVYDKVPNPEDLGVFLE-NEDIRMIRWVGGETAALKQMQHRLAVEYETFCRGSYLPTHGSPD   250
DpCRY/1-534    165   PRPVDDV-------------DLNGVNFGSLPESFYREFVVFDKAPKPEDLGVFLE-NEDIRMIRWVGGETAALKQMQERLAVEYETFCRGSYLPTHGNPD   250
DmCRY1/1-542   172   PRPTADA-------------RLEDATFVELDPEFCRSLKLFEQLPTPEHFNVYGDNMGFLAKINWRGGETQALLLLDERLKVEQHAFERGFYLPNQALPN   258
NbCRY1/1-542   169   PRPVPDP-------------DWHNVKFVKLSEKLIKDLGVFLESPTPEDFSVYPDSLSYLAKVKWIGGETQALLHLDQRLKVEESAFKCGYYLPNQAKPN   255
AgCRY1/1-545   172   PRPVGAP-------------NFEYVEFGRVPALLASELKLCQQMPAPDDFGIHYDGNARIAFQKWIGGETRALEALGARLKQEEAFREGYYLPTQAKPE   258

HsCRY2/1-593   263   LLASPTGLSPYLRFGCLSCRLFYYRLWDLYKKVKRNSTPPLS-------LFGQLLWREFFYTAATNNPRFDRMEGNPICIQIPWDR-NPEALAKWAEGKT   354
MmCRY2/1-592   262   LLASPTGLSPYLRFGCLSCRLFYYRLWDLYKKVKRNSTPPLS-------LFGQLLWREFFYTAATNNPRFDRMEGNPICIQIPWDR-NPEALAKWAEGKT   353
GgCRY2/1-582   253   LLASPTGLSPYLRFGCLSCRLFYYRLWELYKKVKRNSTPPLS-------LYGQLLWREFFYTAATNNPKFDRMEGNPICIQIPWDK-NPEALAKWAEGKT   344
HsCRY1/1-586   244   LLASPTGLSPYLRFGCLSCRLFYFKLTDLYKKVKKNSSPPLS-------LYGQLLWREFFYTAATNNPRFDKMEGNPICVQIPWDK-NPEALAKWAEGRT   335
MmCRY1/1-606   244   LLASPTGLSPYLRFGCLSCRLFYFKLTDLYKKVKKNSSPPLS-------LYGQLLWREFFYTAATNNPRFDKMEGNPICVQIPWDK-NPEALAKWAEGRT   335
ErCRY1/1-620   244   LLASPTGLSPYLRFGCLSCRLFYFKLTDLYKKVKKNSSPPLS-------LYGQLLWREFFYTAATNNPRFDKMEGNPICVQIPWDK-NPEALAKWAEGRT   335
SbCRY1/1-620   244   LLASPTGLSPYLRFGCLSCRLFYFKLTDLYKKVKKNSSPPLS-------LYGQLLWREFFYTAATNNPRFDKMEGNPICVQIPWDK-NPEALAKWAEGRT   335
GgCRY1/1-621   244   LLASPTGLSPYLRFGCLSCRLFYFKLTDLYKKVKKNSSPPLS-------LYGQLLWREFFYTAATNNPRFDKMEGNPICVQIPWDK-NPEALAKWAEGRT   335
- - - - - - - - - - - - - - - - - - - - - - - - - - - - - - - - - - - - - - - - - - - - - - - - - - - - - - - - - - - -
DpCRY2/1-742   270   LLSSQTGLSPYLRFGCLSTRLFYYQLSELYKRIKQ-ERPPLS-------LHGQILWREFFYCAATRNPNFDRMEGNPICVQIPWEK-NQEALKKWANGQT   360
MbCRY1/1-548   251   LLGPPISLSPALRFGCLSVRSFYWSVQDLFRQVHQG---RLATQSASHFITGQLIWREYFYTMSVNNPNYGQMAGNPICLDIPWKEPEDELQRWVEGRT   347
HaCRY1/1-528   251   LLGPPISLSPALRFGCLSVRSFYWAVQDLFRQVHQG---RLTTNSASHFITGQLIWREYFYTMSVNNPNYGQMAGNPICLDIPWKNPEGDELQRWVEGRT   347
BmCRY1/1-536   251   LLGPPISLSPALRFGCLSVRKFYWSLQDLFQQVHQG---SLCS---TQYITGQLIWREYFYTMSVNNPHYGQMTDNPICLDIPWKSPEGDELERWASGRT   344
DpCRY/1-534    251   LLGPPISLSPALRFGCLSVRRFYWSLQDLFQQVHQG---RLAS---TQFITGQLIWREYFYTMSVNNPNYAQMSGNPICLDIPWKEPENDELQRWKEGRT   344
DmCRY1/1-542   259   IHDSPKSMSAHLRFGCLSVRRFYWSVHDLFKNVQLRACVRGVQMTGGAHITGQLIWREYFYTMSVNNPNYDRMEGNDICLSIPWAKPNENLLQSWRLGQT   358
NbCRY1/1-542   256   ILESPKSMSAHLRFGCLSVRKFYWDVHDLFKDVQQQAECLGMLMSGGAHITGQLIWREYFYTMSVNNPNYDRMEGNEICLNIPWAETNHIQLQRWTQGKT   355
AgCRY1/1-545   259   ILGPATSMSAALRFGCLSVRMFYWCVHDLFAKVQSNSQFK---YPGGHHITGQLIWREYFYTMSVQNPHYGEMERNPICLNIPWYKPEDDSLTRWKEGRT   355
```

Figure S11. Sequence alignment of Type I and Type II CRY (part 1).

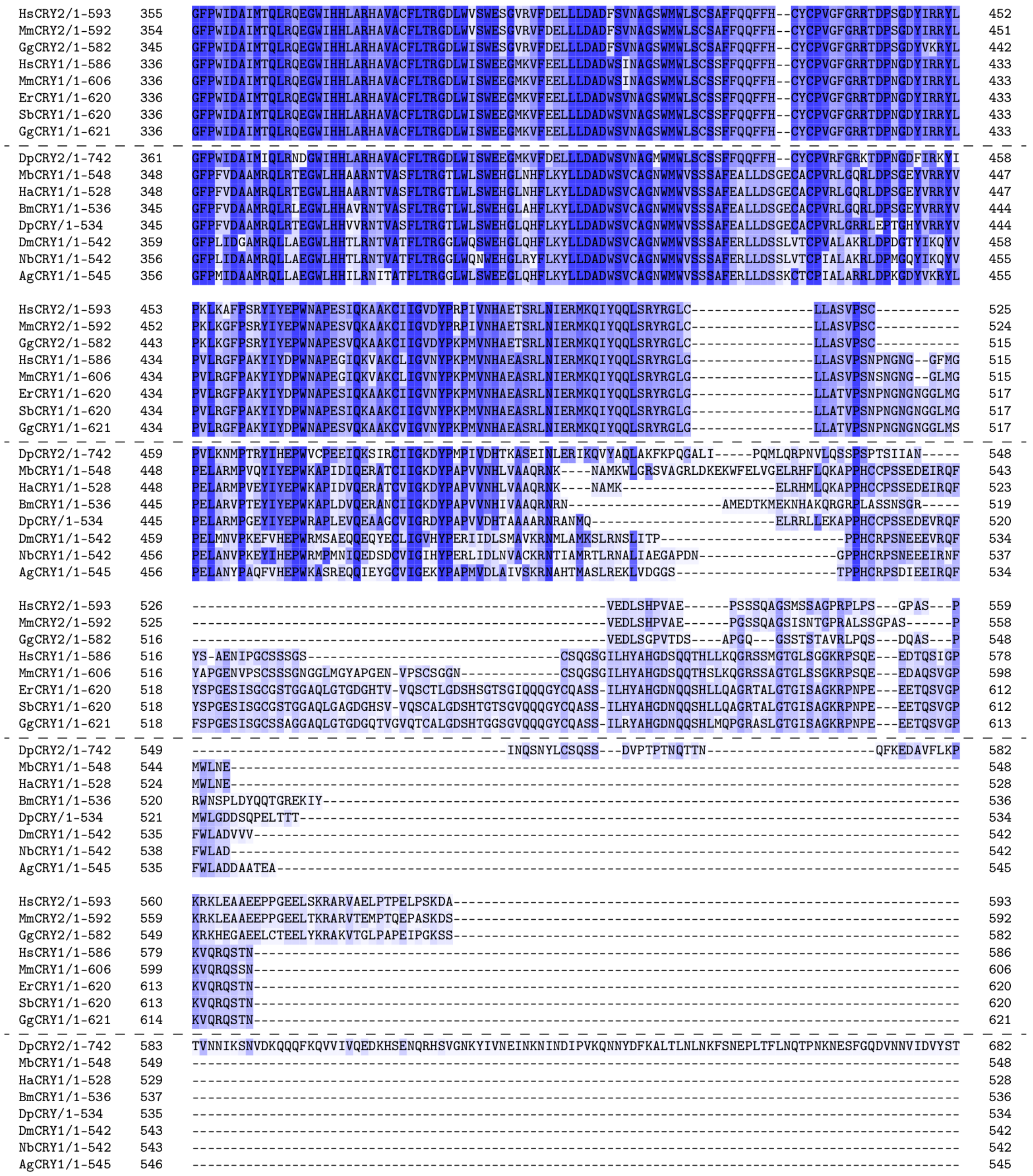

Figure S11 (continued). Sequence alignment of Type I and Type II CRY (part 2).

```
HsCRY2/1-593   594   ------------------------------------------------------------   593
MmCRY2/1-592   593   ------------------------------------------------------------   592
GgCRY2/1-582   583   ------------------------------------------------------------   582
HsCRY1/1-586   587   ------------------------------------------------------------   586
MmCRY1/1-606   607   ------------------------------------------------------------   606
ErCRY1/1-620   621   ------------------------------------------------------------   620
SbCRY1/1-620   621   ------------------------------------------------------------   620
GgCRY1/1-621   622   ------------------------------------------------------------   621
- - - - - - - - - - - - - - - - - - - - - - - - - - - - - - - - - - - - - - - - - - - - -
DpCRY2/1-742   683   SKPKFYFTDNGVITHNENAQTFKRDSYSDNYNKESTGSNRVGEVHSNNPQTDKISSEKKN   742
MbCRY1/1-548   549   ------------------------------------------------------------   548
HaCRY1/1-528   529   ------------------------------------------------------------   528
BmCRY1/1-536   537   ------------------------------------------------------------   536
DpCRY/1-534    535   ------------------------------------------------------------   534
DmCRY1/1-542   543   ------------------------------------------------------------   542
NbCRY1/1-542   543   ------------------------------------------------------------   542
AgCRY1/1-545   546   ------------------------------------------------------------   545
```

Figure S11 (continued). Sequence alignment of Type I and Type II CRY (part 3). Sequence alignment between eight Type I and eight Type II CRY. Colour coding: The darker the underlying blue the higher the sequence similarity within the chosen sixteen sequences. Abbreviations: Hs = *Homo sapiens* (human), Mm = *Mus musculus* (house mouse), Gg = *Gallus gallus* (chicken), Er = *Erithacus rubecula* (European robin), Sb = *Sylvia borin* (garden warbler), Dp = *Danaus plexippus* (monarch butterfly), Mb = *Mamestra brassicae* (cabbage moth), Ha = *Helicoverpa armigera* (cotton bollworm), Bm = *Bombyx mori* (domesticated silkmoth), Dm = *Drosophila melanogaster* (fruit fly), Nb = *Neobellieria bullata* (grey flesh fly), Ag = *Anopheles gambiae* (mosquito).

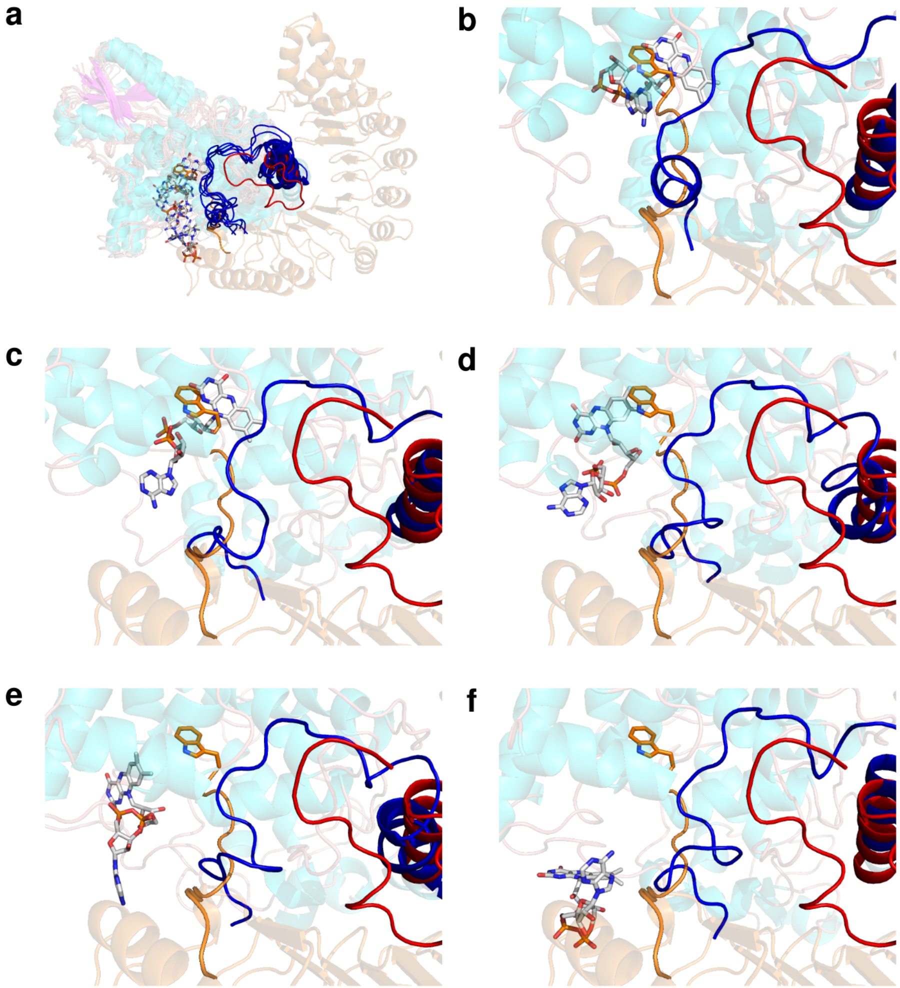

Figure S12. Comparison between MmCRY2 structures from metadynamics simulations with the MmCRY2/FBLX3-Complex. a: Overlay between the MmCRY2/FBLX3-complex (PDB code: 4I6J; 7 amino acids at the C-terminus are not resolved) with the five snapshots (**b-f**) from the metadynamics simulations at M0 to M4 from Figure 2 (58 amino acids are missing in our model since these are not resolved). **b-f**: Zoom of the FAD-binding pocket for each individual snapshot M0 (**b**) to M4 (**f**). Colour coding: FBXL3: orange; C-terminus of FBXL3: red; MmCRY2: cyan; C-terminus of MmCRY2: blue; FAD: highlighted with white carbon atoms.

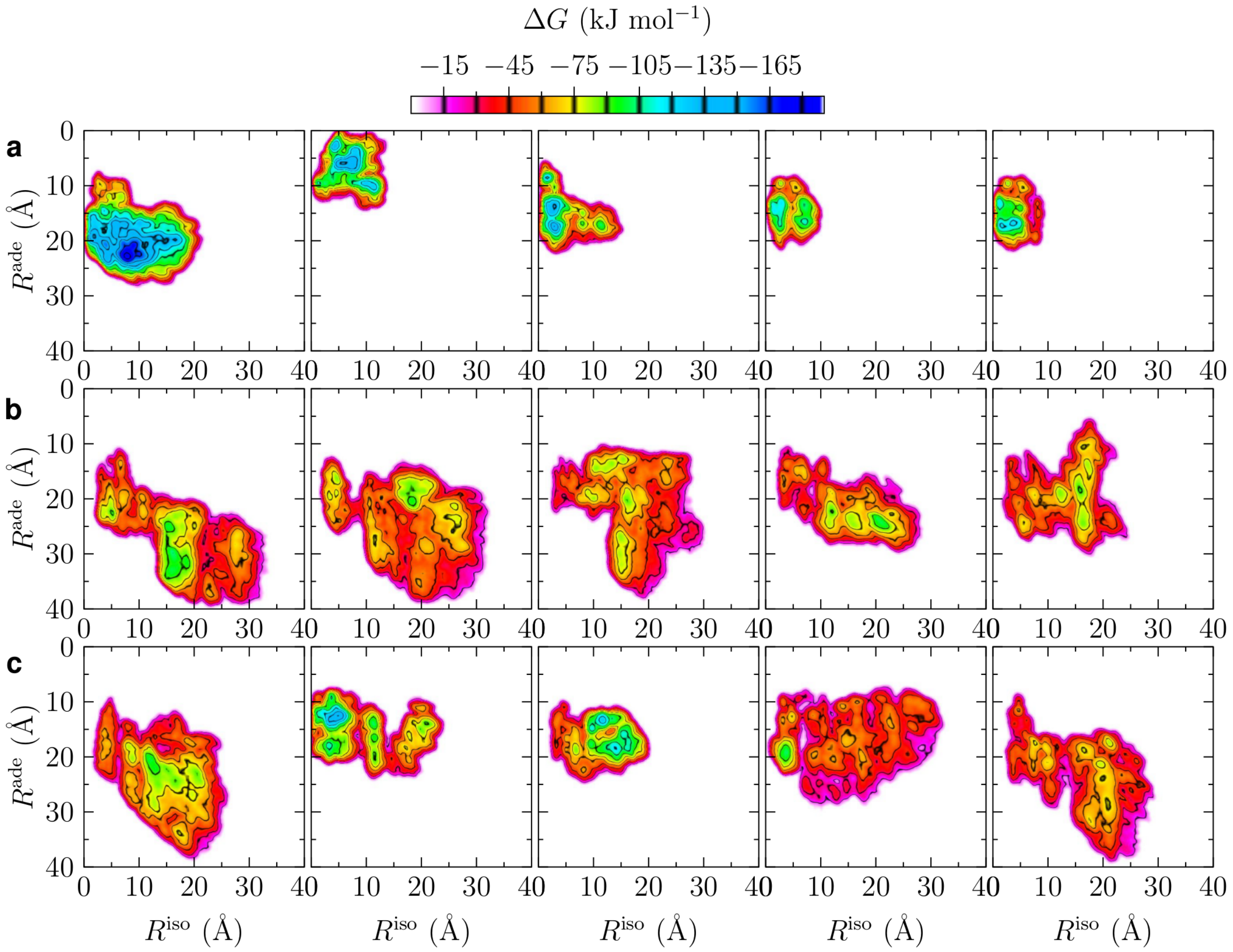

Figure S13. FAD-binding studies based on metadynamics simulations. Five individual 2-dimensional free energy (ΔG) plots for FAD (un)binding from 100 ns MTD simulations for each of DmCRY (**a**), MmCRY2 (**b**) and SbCRY1a (**c**). R^{ade} is the distance between the centre-of-masses of the adenine and the binding pocket residues, and R^{iso} the distance between the centre-of-masses of the isoalloxazine and the binding pocket residues as shown in Figure S4.

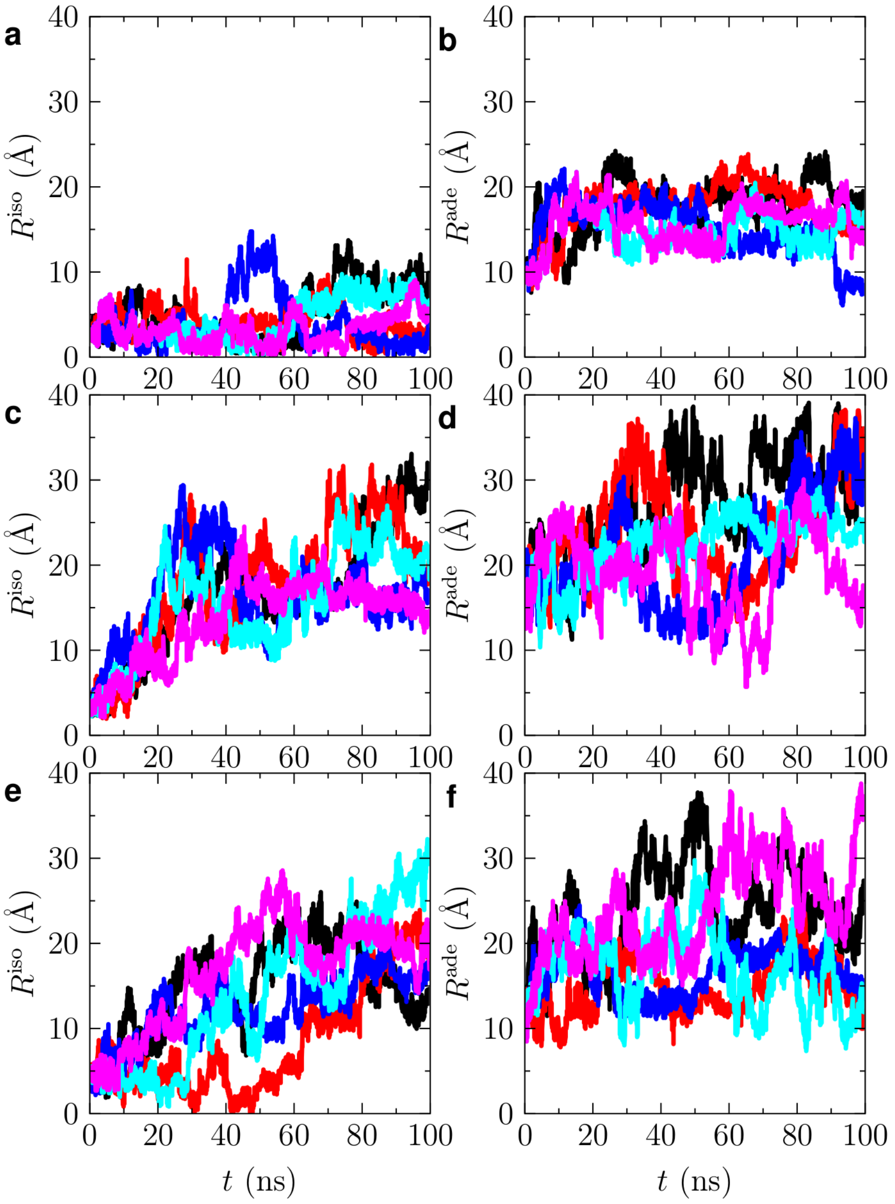

Figure S14. FAD-binding studies based on metadynamics simulations. Plotted are the values of the CVs, R^{iso} and R^{ade}, for each individual of five simulations over 100 ns for the systems DmCRY (**a** and **b**), MmCRY2 (**c** and **d**), and SbCRY1a (**e** and **f**).

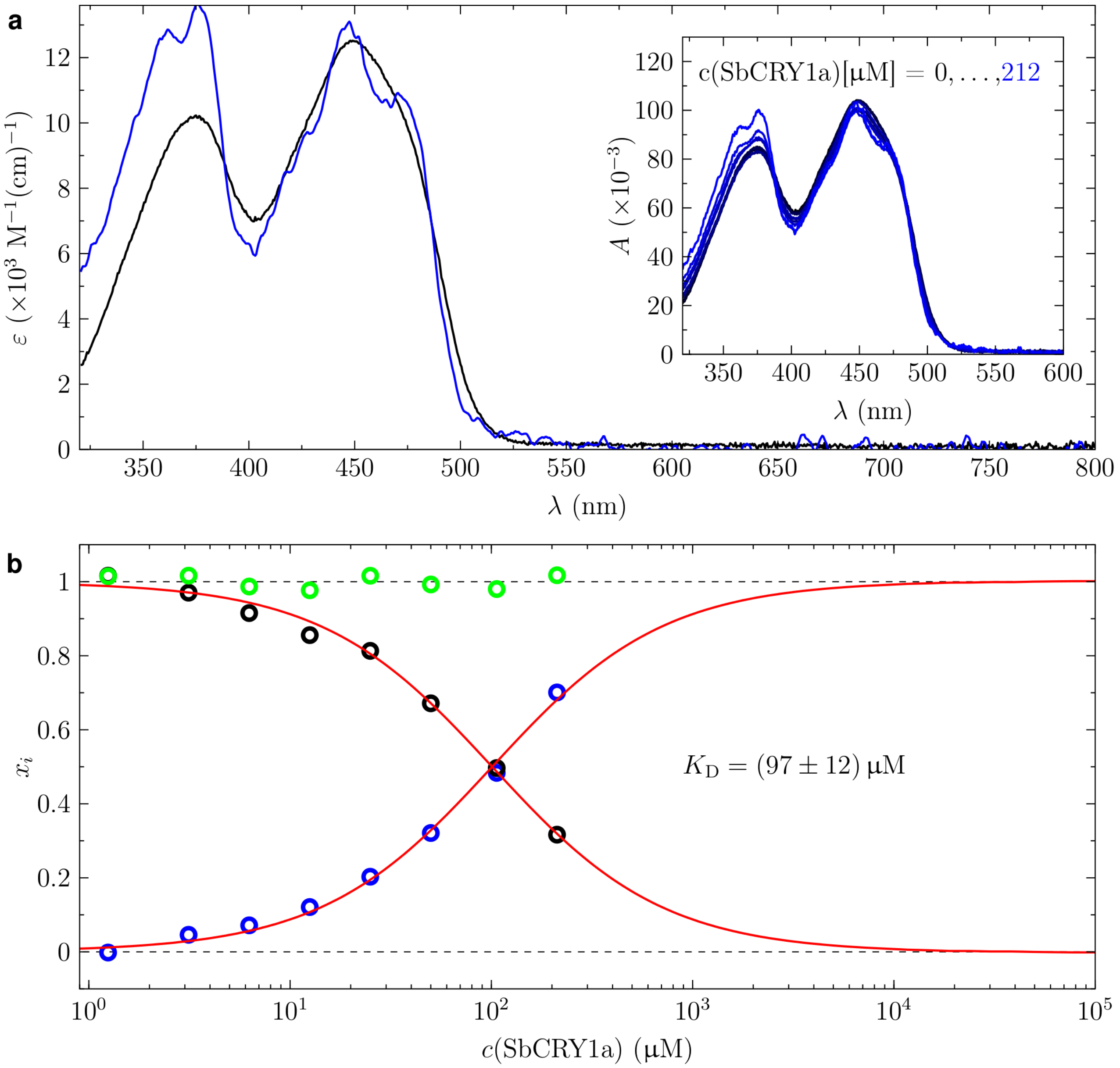

Figure S15. Determination of the FAD-binding affinity in SbCRY1a. a: pure UV-visible absorption spectra of unbound FAD (black) and bound in SbCRY1a (blue) extracted from the raw data (inset in panel **a**: raw data minus fitted scatter contribution) as described in the Materials and Methods section of the main text. **c**: mole fractions of unbound FAD (black open circles) and bound in SbCRY1a (blue open circles) as determined from fitting the pure spectra from panel **a** to each spectrum of the inset in panel **a** correlated with the SbCRY1a-apo concentration. The green open circles represent the sum of the mole fractions. The red lines represent the global fits of the binding equation 4 to the data.

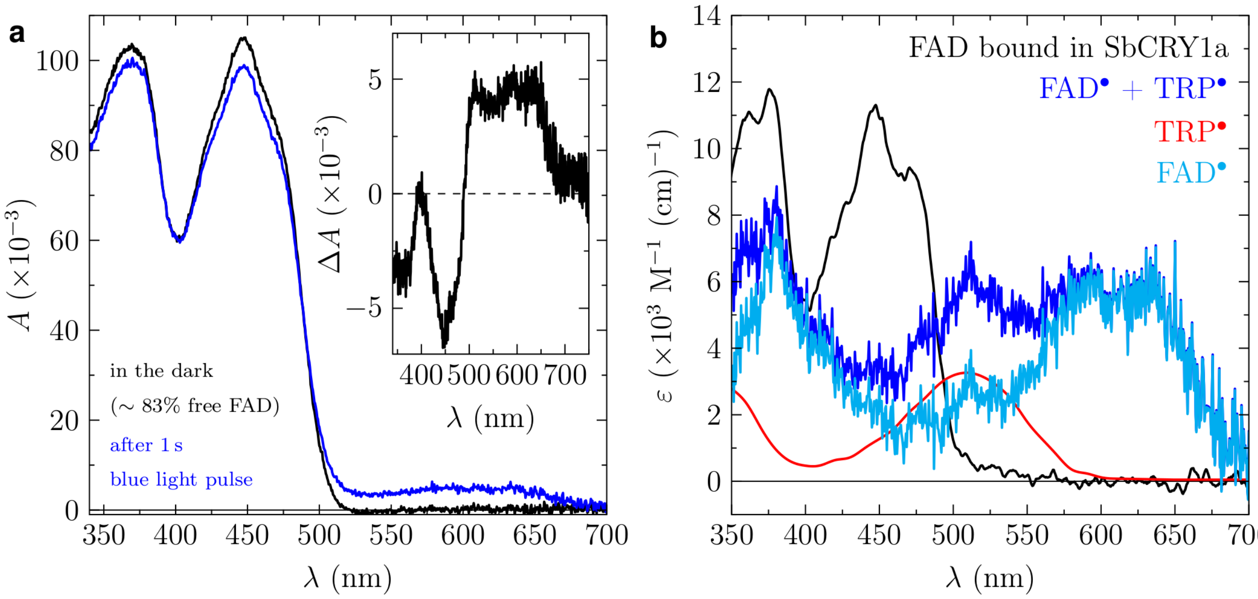

Figure S16. Determination of the pure neutral FAD radical spectrum and the counter neutral tryptophan radical in SbCRY1a *via in silico* titration. Absorption spectra of a sample containing 8 µM FAD and *ca*. 33.8 µM SbCRY1a before (black line in panel **a**) and directly after illumination with a 1 s blue-light pulse (blue line in panel **a**). Since this sample still contained *ca*. 13% of unbound FAD, the difference spectrum (inset in panel **a**) subtracts the contribution of unbound FAD. Adding the pure spectrum of fully bound FAD to SbCRY1a from Figure S15**a** to the difference spectrum in such a way that its characteristic fine structural features disappear, results in a spectrum (blue line in panel **b**) with a high similarity to known spectra for the neutral flavin semiquinone radical, *e.g.* shown in[1,2]. However, some deviations can still be seen especially in the range between 450 to 550 nm. These deviations can be explained by the contributions of the neutral tryptophan radical (red line extracted from[3]). By subtracting a certain amount of the neutral tryptophan radical from the mixture spectrum it is possible to obtain a perfect match to the known flavin neutral radical spectrum (cyan line). (A detailed time-resolved analysis of the mechanism including branching ratios, the photoproduct, and a discussion about the proton donor is beyond the scope of this work and will be presented elsewhere.)

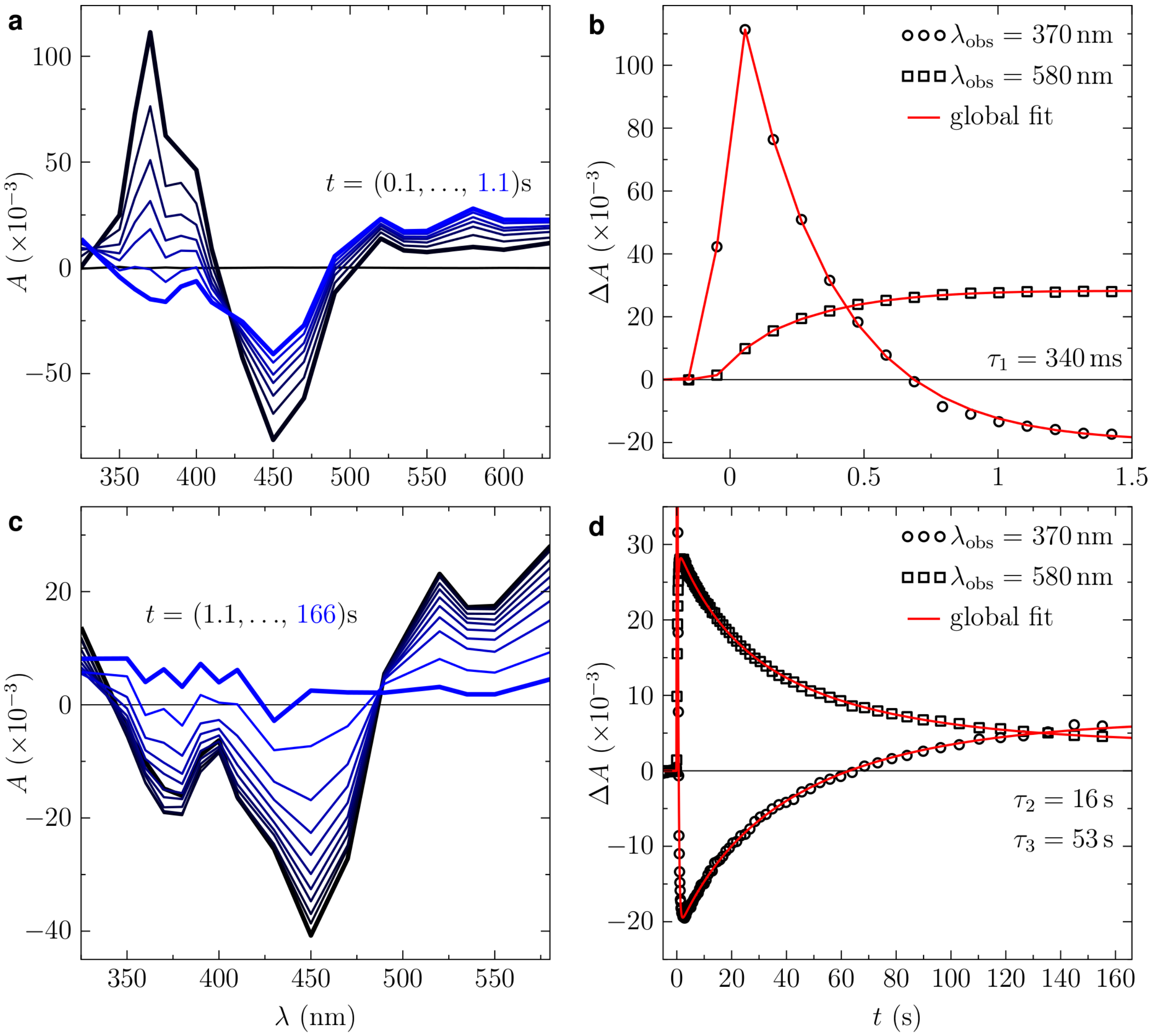

Figure S17. Photo-induced formation and decay of the neutral FAD semiquinone radical bound in SbCRY1a. UV-visible absorption changes at single wavelengths in the range from 350 to 580 nm induced by single 100 ms blue-light pulse were recorded a sample containing 27.4 µM FAD and *ca.* 690 µM SbCRY1a. The spectrally (**a** and **c**) and temporally (**b** and **d**) resolved UV-visible absorption changes up to 1 s (**a** and **b**) and 160 s (**c** and **d**) are shown at two selected wavelength representative for the main features of the anionic (370 nm) and neutral (580 nm) FAD semiquinone radicals. The red lines represent the global fit of two exponential functions to the entire data matrix with the lifetimes of 340 ms, 16 s, and 53 s. (A detailed time-resolved analysis of the mechanism including branching ratios, the photoproduct, and a discussion about the proton donor is beyond the scope of this work and will be presented elsewhere.)

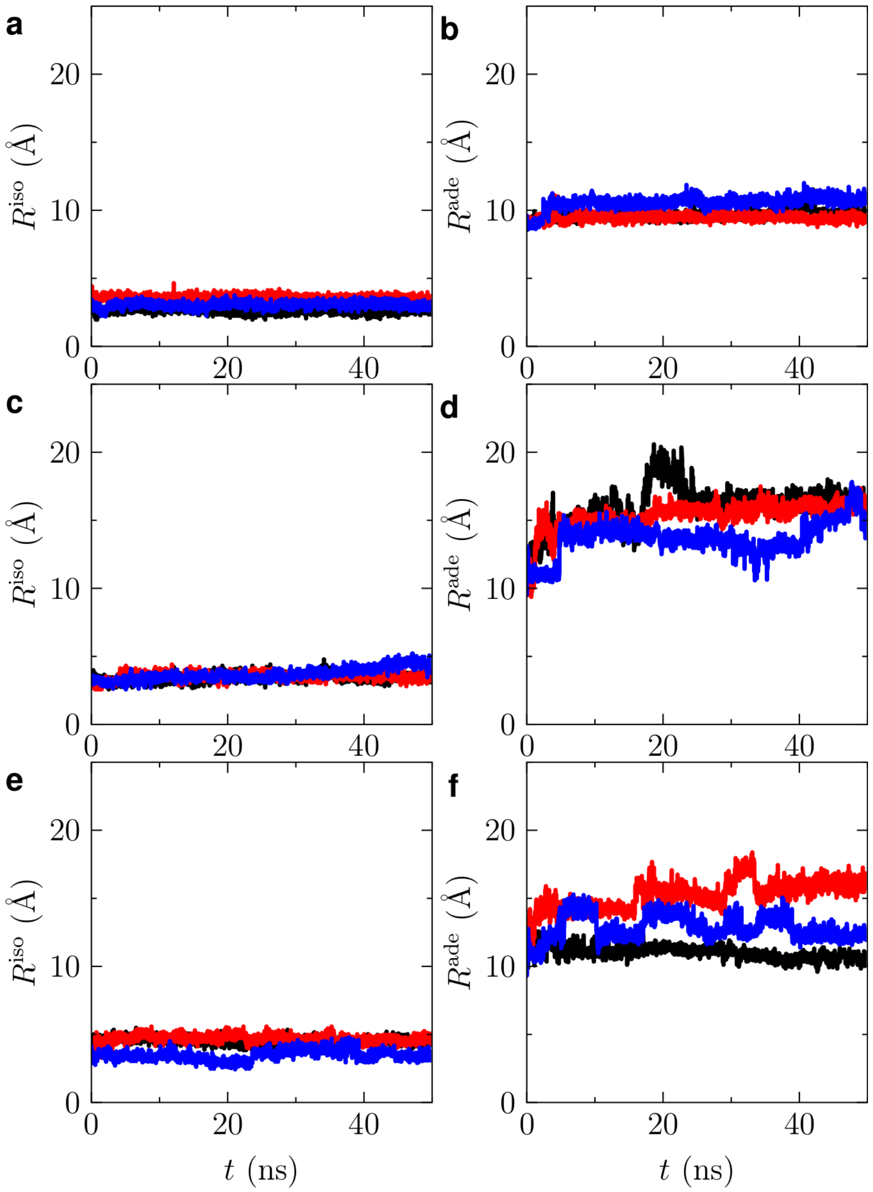

Figure S18. Movement of FAD during conventional MD simulations. Plots of R^{iso} and R^{ade} for DmCRY (**a**,**b**), MmCRY2 (**c**,**d**) and SbCRY1a (**e**,**f**) from 3 MD simulations using different seeds for initial velocity generation. While the FAD remains bound during each of these short simulations, the adenine is clearly more tightly bound in DmCRY, in accord with the structural differences outlined in Figure 3.

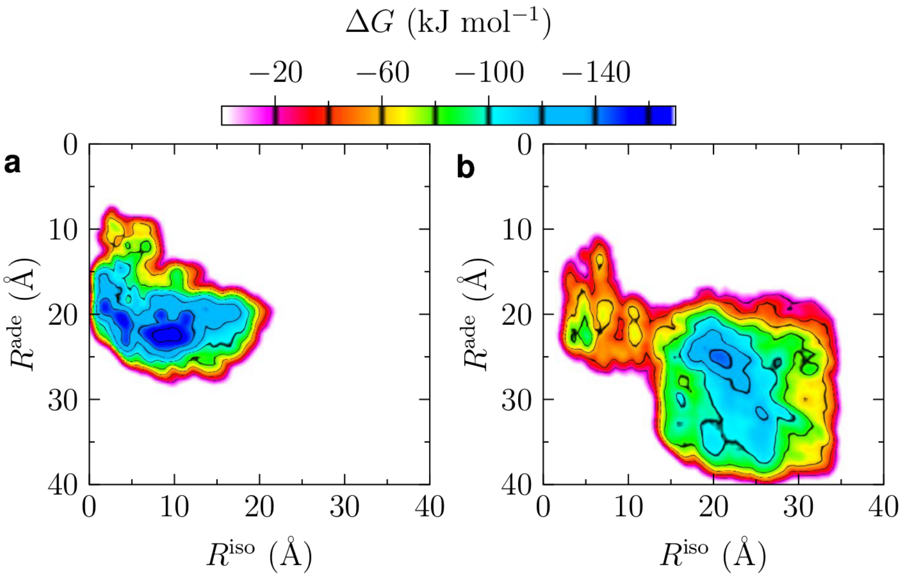

Figure S19. FAD-binding studies based on metadynamics simulations. 2-dimensional free energy (ΔG) plots for FAD (un)binding from 300 ns MTD simulations for DmCRY (**a**) and MmCRY2 (**b**). R^{ade} is the distance between the centre-of-masses of the adenine and the binding pocket residues, and R^{iso} the distance between the centre-of-masses of the isoalloxazine and the binding pocket residues as shown in Figure S9.

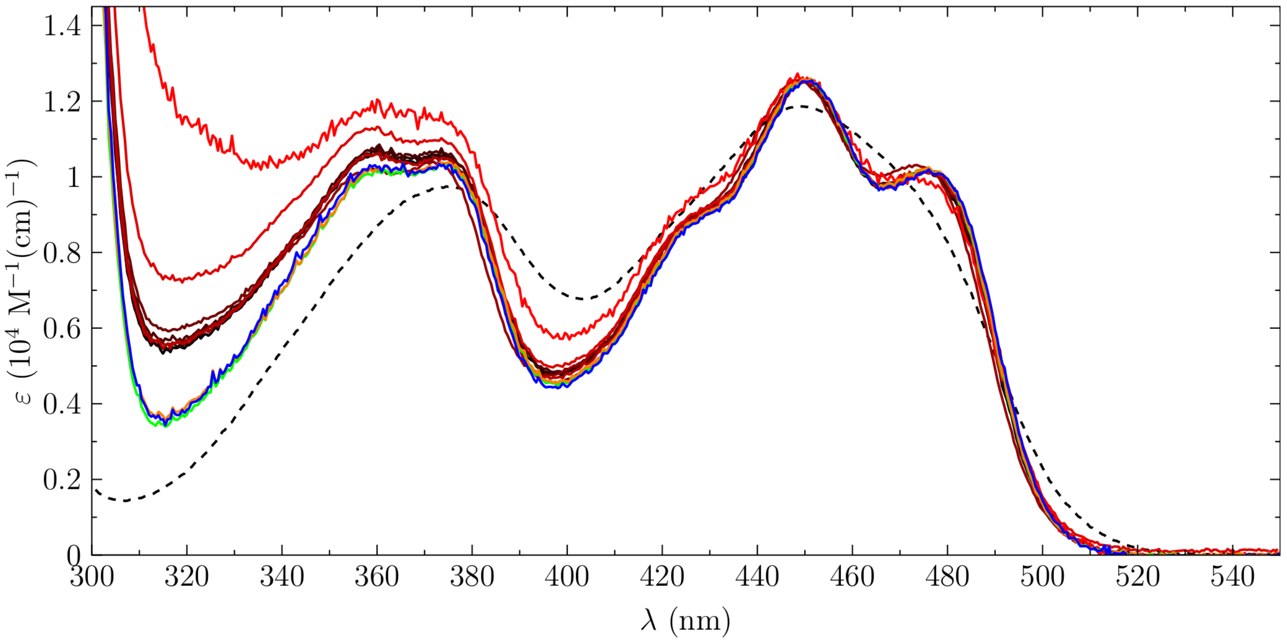

Figure S20. UV-visible absorption of DmCRY wild type, variants and free FAD. Zoom of Figure 3**c** in the spectral range from 300 to 550 nm. The colour coding corresponds to the inset in Figure 3**c**. The additional colours correspond to spectra of the following mutants: Q311A (green), R298Q-Q311A (orange), and R237Q-Q311A (blue). The extinction coefficients are scaled to the absorbance maximum of FAD at 450 nm. The additional variants resulted in no effect for the single variant, but in a loss in FAD binding affinity at maximum for the variant R237Q-Q311A, of ca. 8%. The small effect can be explained most likely due to the H-bonding ability of the glutamine, which somewhat restores − not to the same extent as the wild type – the interaction to the FAD pyrophosphate. However, this variant does not introduce new electrostatic interactions and, thus, a disruption of the whole protein structural conformation is less likely demonstrating the importance of the discussed amino acids necessary for FAD binding. Since these variants as well as the double variant R298E-Q311E show almost identical fine structures in their UV/Vis absorption spectra due to bound FAD, one can assume that the interaction site for the isoalloxazine ring is comparable/identical to wild type.

References

1. Kutta, R. J., Kensy, U. & Dick, B. Measuring photochemical intermediates with a streak camera - a new technique for transient absorption. in *Chemical Photocatalysis* (2013).
2. Lanzl, K., Sanden-Flohe, M. v., Kutta, R. J. & Dick, B. Photoreaction of mutated LOV photoreceptor domains from *chlamydomonas reinhardtii* with aliphatic mercaptans: implications for the mechanism of wild type LOV. *PCCP* **12**, 6594–6604 (2010).
3. Solar, S., Getoff, N., Surdhar, P. S., Armstrong, D. A. & Singh, A. Oxidation of tryptophan and N-methylindole by $N_3^{\bullet}$, Br_2^{-}, and $(SCN)_2^{-}$ radicals in light- and heavy-water solutions: a pulse radiolysis study. *J. Phys. Chem.* **95**, 3639–3643 (1991).
